# Integrated transcriptome, small RNA and degradome sequencing approaches provide insights into Ascochyta blight resistance in chickpea

**DOI:** 10.1111/pbi.13026

**Published:** 2018-12-01

**Authors:** Vanika Garg, Aamir W. Khan, Himabindu Kudapa, Sandip M. Kale, Annapurna Chitikineni, Sun Qiwei, Mamta Sharma, Chuanying Li, Baohong Zhang, Liu Xin, P.B. Kavi Kishor, Rajeev K. Varshney

**Affiliations:** ^1^ Center of Excellence in Genomics & Systems Biology (CEGSB) International Crops Research Institute for the Semi‐Arid Tropics (ICRISAT) Patancheru Telangana India; ^2^ Department of Genetics Osmania University Hyderabad Telangana India; ^3^ BGI‐Shenzhen Shenzhen China; ^4^ Integrated Crop Management ICRISAT Patancheru Telangana India; ^5^ Department of Biology East Carolina University Greenville NC USA

**Keywords:** miRNA, differentially expressed genes, fungal stress, mRNA targets, Ascochyta blight resistance, miRNA‐mRNA, cleavage

## Abstract

Ascochyta blight (AB) is one of the major biotic stresses known to limit the chickpea production worldwide. To dissect the complex mechanisms of AB resistance in chickpea, three approaches, namely, transcriptome, small RNA and degradome sequencing were used. The transcriptome sequencing of 20 samples including two resistant genotypes, two susceptible genotypes and one introgression line under control and stress conditions at two time points (3rd and 7th day post inoculation) identified a total of 6767 differentially expressed genes (DEGs). These DEGs were mainly related to pathogenesis‐related proteins, disease resistance genes like NBS‐LRR, cell wall biosynthesis and various secondary metabolite synthesis genes. The small RNA sequencing of the samples resulted in the identification of 651 miRNAs which included 478 known and 173 novel miRNAs. A total of 297 miRNAs were differentially expressed between different genotypes, conditions and time points. Using degradome sequencing and *in silico* approaches, 2131 targets were predicted for 629 miRNAs. The combined analysis of both small RNA and transcriptome datasets identified 12 miRNA‐mRNA interaction pairs that exhibited contrasting expression in resistant and susceptible genotypes and also, a subset of genes that might be post‐transcriptionally silenced during AB infection. The comprehensive integrated analysis in the study provides better insights into the transcriptome dynamics and regulatory network components associated with AB stress in chickpea and, also offers candidate genes for chickpea improvement.

## Introduction

Chickpea (*Cicer arietinum* L.) is one of the most widely grown legume crops with an annual global production of ~12.09 million tons (FAO, 2016). It is a self‐pollinated, diploid annual crop grown mostly in arid and semi‐arid regions of the world. It is a rich source of proteins and essential amino acids for millions of people living in developing countries. Along with substantial nutritive value, like other legume crops, chickpea crop increases soil fertility by fixing atmospheric nitrogen. Owing to the high nutritional and commercial importance of chickpea, several research efforts have been carried out in recent past to increase its production. However, chickpea production is far below its demand and has not achieved its potential yield owing to major constraints in the form of several biotic stresses like Ascochyta blight, Fusarium wilt and abiotic stresses like drought, salinity, heat. Though earlier efforts have considerably enhanced chickpea productivity, there is still a need to take more rigorous steps towards chickpea improvement to meet its demand.

Ascochyta blight (AB), caused by fungus *Ascochyta rabiei,* is an important foliar disease of chickpea. AB is necrotrophic in nature and is rampant under cool and humid weather conditions. AB infection usually leads to necrotic lesions on leaves, stem breakage, pod abortion and eventually the plant death (Pande *et al*., 2005). The severity of the disease is manifested in the form of 100% yield loss under favorable conditions. The occurrence of AB has been reported from more than 40 countries across the globe (Sharma and Ghosh, 2016). The management of AB relies upon an integrated approach that includes intensive fungicide application, crop rotation strategies, seed treatment and use of resistant cultivars. However, the use of varieties resistant to AB remains the most cost‐effective and environmentally sustainable solution to tackle AB. The emergence of *A. rabiei* virulent isolates with low sensitivity towards fungicides further adds to the need for development of AB resistant chickpea cultivars (Sharma and Ghosh, 2016). With an objective to develop AB resistant cultivars through molecular breeding, a number of QTLs for AB resistance on various linkage groups have been reported (Li *et al*., 2017a; Sabbavarapu *et al*., 2013). Furthermore, seven introgression lines for two QTLs conferring resistance to AB (ABQTL‐I and ABQTL‐II) have been developed through marker‐assisted backcrossing (Varshney *et al*., 2014). However, to fasten the molecular breeding process and tackle the issue of possible evolution of AB pathogen(s), it is important to have the deeper knowledge of AB resistance mechanisms in chickpea.

Disease resistance is a complex phenomenon involving numerous mechanisms. When under stress, plants develop a myriad of mechanisms for sensing and adapting to the environmental changes through a network of genetic regulations. These genetic regulations include alteration in gene expression at both transcriptional and post‐transcriptional levels, resulting in changes in signal transduction pathways and other metabolic processes. Several approaches such as microarrays, EST sequencing and RNA sequencing (RNA‐seq) have been used to study these genetic regulations. However, RNA‐seq facilitates the transcript identification and gene expression quantification in a robust way and has been widely used to understand the transcriptional dynamics in plants. In the case of chickpea, several transcriptome studies have been undertaken to identify key abiotic stress‐responsive genes for tolerance to drought and salinity (Garg *et al*., 2016; Mashaki *et al*., 2018). In contrast, for identification of biotic stress‐responsive genes in chickpea, a few studies have been reported (Jain *et al*., 2015; Upasani *et al*., 2017). In case of AB stress mechanism, a few gene expression studies have been performed, mainly using 768‐features expression arrays (Coram and Pang, 2006; Mantri *et al*., 2010), however, a genome‐wide and comprehensive analysis of genes involved in AB resistance is not yet available.

It is well studied that epigenetic regulations in the form of small non‐coding RNAs are known to alter the gene expression. Small RNAs such as microRNAs (miRNAs) are 20–24 nucleotide (nt) endogenous non‐coding RNAs derived from single‐stranded stem‐loop precursors. In plants, miRNAs not only control the post‐transcriptional regulation of their targets but also interact with each other in regulatory networks affecting development and responses to biotic and abiotic stresses. miRNAs control the expression of genes in spatial and temporal‐specific manner in response to stress. A number of studies have suggested the importance of miRNAs in plant defense response (Kohli *et al*., 2014; Sarkar *et al*., 2017). Although miRNAs have been extensively studied in plants, their regulatory mechanism is still unclear on miRNA‐mediated response to biotic stress. For understanding the miRNA responses, the accurate and confident prediction of targets is indispensable. Plant miRNAs are known to have perfect or near‐perfect complementarity with their targets and can be predicted using both computational tools and sequencing approaches like degradome analysis. Recently, a few studies on the integrated analysis of mRNA and miRNA expression profiles have been published (Cao *et al*., 2017; Guo *et al*., 2017; Sarkar *et al*., 2017). For instance, an extensive interaction analysis of mRNA and miRNA uncovered the molecular interactions between pathogen *Potato virus Y* and tobacco (Guo *et al*., 2017). Similarly, another study identified important regulators of Alternaria‐stress response in tomato (Sarkar *et al*., 2017).

With an objective to get deeper insights into the AB resistance mechanisms in chickpea as well as to understand molecular interactions between *A. rabiei* and chickpea, the present study employed transcriptome, small RNA and degradome sequencing approaches using diverse chickpea genotypes including two susceptible genotypes, two resistant genotypes and an introgression line carrying two AB‐resistance QTLs. A comprehensive and integrated analysis of these different datasets have identified differentially expressed genes (DEGs), miRNAs, their targets and also delineated the interplay between all these components in chickpea in response to AB.

## Results

### Transcriptome sequencing

In the study, 20 samples representing two moderately resistant genotypes (ICCV 05530 and ILC 3279), two susceptible genotypes (C 214 and Pb 7) and one introgression line showing resistance to AB (BC_3_F_6_), under control (non‐inoculated) and stress (AB inoculated) conditions at two time points [3rd and 7th day post inoculation (dpi)] were sequenced. A total of 1350.1 million reads were generated from the paired‐end sequencing of these 20 samples. After the stringent quality filters, 96.7% (1305.2 million) of the reads representing high‐quality reads were processed for further analysis. These reads were mapped on the chickpea genome sequence. On an average, about 93% (1208.2 million) of the high‐quality reads were mapped to the chickpea genome. The sequencing data and mapping statistics reflect a very high‐quality transcriptome sequencing. The sample‐wise details of sequence data generated, filtered reads and reads mapped on the genome are given in Table 1. A reference‐guided assembly of the mapped reads using Cufflinks–Cuffmerge pipeline identified a total of 31 459 genes. For ease of understanding, the samples have been designated as genotype‐condition‐time point. For example, the sample ILC 3279‐C‐3d denotes genotype ILC 3279 in control condition at 3rd dpi and ILC 3279‐S‐7d denotes genotype ILC 3279 under stress condition at 7th dpi.

**Table 1:** Summary of transcriptome and small RNA sequencing data generated for 20 samples using Illumina sequencing platform

Sample	Transcriptome sequencing			Small RNA sequencing							
	Raw reads	High‐quality reads (Q20)	Uniquely mapped reads	Raw reads	High‐quality reads (Q20)	Reads <18 or >35 nt	Repeat reads	rRNA reads	tRNA reads	snoRNA reads	miRNA reads
BC_3_F_6_‐C‐3d	60 749 026	58 307 286	53 925 123	34 541 167	33 788 438	5 580 073	18 940 014	3 231 640	410 549	13 629	704 514
BC_3_F_6_‐C‐7d	70 143 768	68 035 056	63 282 216	22 643 884	22 180 985	10 116 568	7 858 455	783 815	146 372	10 521	264 770
BC_3_F_6_‐S‐3d	67 510 428	65 028 960	59 715 937	26 974 790	26 301 095	1 229 051	16 089 121	2 925 903	303 676	23 025	706 916
BC_3_F_6_‐S‐7d	68 727 054	66 718 240	61 029 562	26 470 944	25 146 500	1 915 689	13 676 356	5 201 517	456 734	21 767	494 801
C 214‐C‐3d	61 050 574	59 154 326	55 109 324	22 854 828	22 225 437	1 522 720	10 067 883	3 250 766	1 422 273	10 490	337 310
C 214‐C‐7d	56 480 126	53 942 318	49 588 638	22 070 578	20 933 738	1 153 972	14 526 247	1 438 068	281 500	14 580	558 848
C 214‐S‐3d	71 989 730	69 824 680	64 680 734	28 023 898	27 680 554	3 747 207	17 484 491	1 874 295	274 061	10 887	795 241
C 214‐S‐7d	54 366 940	52 685 694	48 200 639	25 493 856	24 376 962	2 090 016	14 421 146	3 648 744	354 864	17 654	523 387
ICCV 05530‐C‐3d	62 478 856	60 571 968	56 135 229	23 472 528	23 065 108	1 379 015	14 068 233	2 474 119	345 157	7008	685 411
ICCV 05530‐C‐7d	61 582 488	59 373 280	55 193 397	26 808 926	26 211 017	1 954 666	16 533 251	2 406 505	389 040	10 385	793 820
ICCV 05530‐S‐3d	72 688 564	70 523 506	65 673 033	27 114 190	26 651 176	6 261 321	13 657 927	2 112 152	418 415	9621	549 230
ICCV 05530‐S‐7d	60 137 372	58 124 704	53 907 489	30 629 458	29 368 612	2 184 853	20 559 115	2 109 075	270 702	23 633	634 595
ILC 3279‐C‐3d	64 575 072	61 999 362	57 803 426	23 345 383	22 849 072	4 501 471	12 642 469	1 495 647	262 078	7346	589 180
ILC 3279‐C‐7d	76 446 674	73 443 334	68 266 373	27 087 841	26 408 530	1 382 123	15 514 413	4 573 745	1 159 799	26 461	464 350
ILC 3279‐S‐3d	72 895 320	70 857 522	66 052 608	26 789 897	26 341 109	3 771 272	16 144 324	2 702 064	420 439	11 167	530 720
ILC 3279‐S‐7d	89 086 896	86 289 642	80 162 092	34 242 425	32 843 585	2 712 995	21 536 934	2 320 354	564 990	43 003	615 380
Pb 7‐C‐3d	74 253 380	72 194 662	67 235 029	24 407 891	23 917 009	3 709 743	14 481 335	1 702 670	274 383	6709	446 905
Pb 7‐C‐7d	68 789 242	66 062 580	60 745 741	25 950 674	24 510 418	1 945 959	16 856 448	2 774 137	317 118	12 515	411 616
Pb 7‐S‐3d	63 411 320	61 656 214	56 936 881	23 748 014	23 308 958	985 519	16 104 515	2 059 807	207 396	16 776	500 210
Pb 7‐S‐7d	72 782 344	70 378 552	64 606 941	30 137 829	29 084 571	1 923 999	17 582 076	4 041 252	488 877	19 078	673 225
Total	1 350 145 174	1 305 171 886	1 208 250 412	532 809 001	517 192 874	60 068 232	308 744 753	53 126 275	8 768 423	316 255	11 280 429

#### Differential gene expression analysis

**Figure 1:**
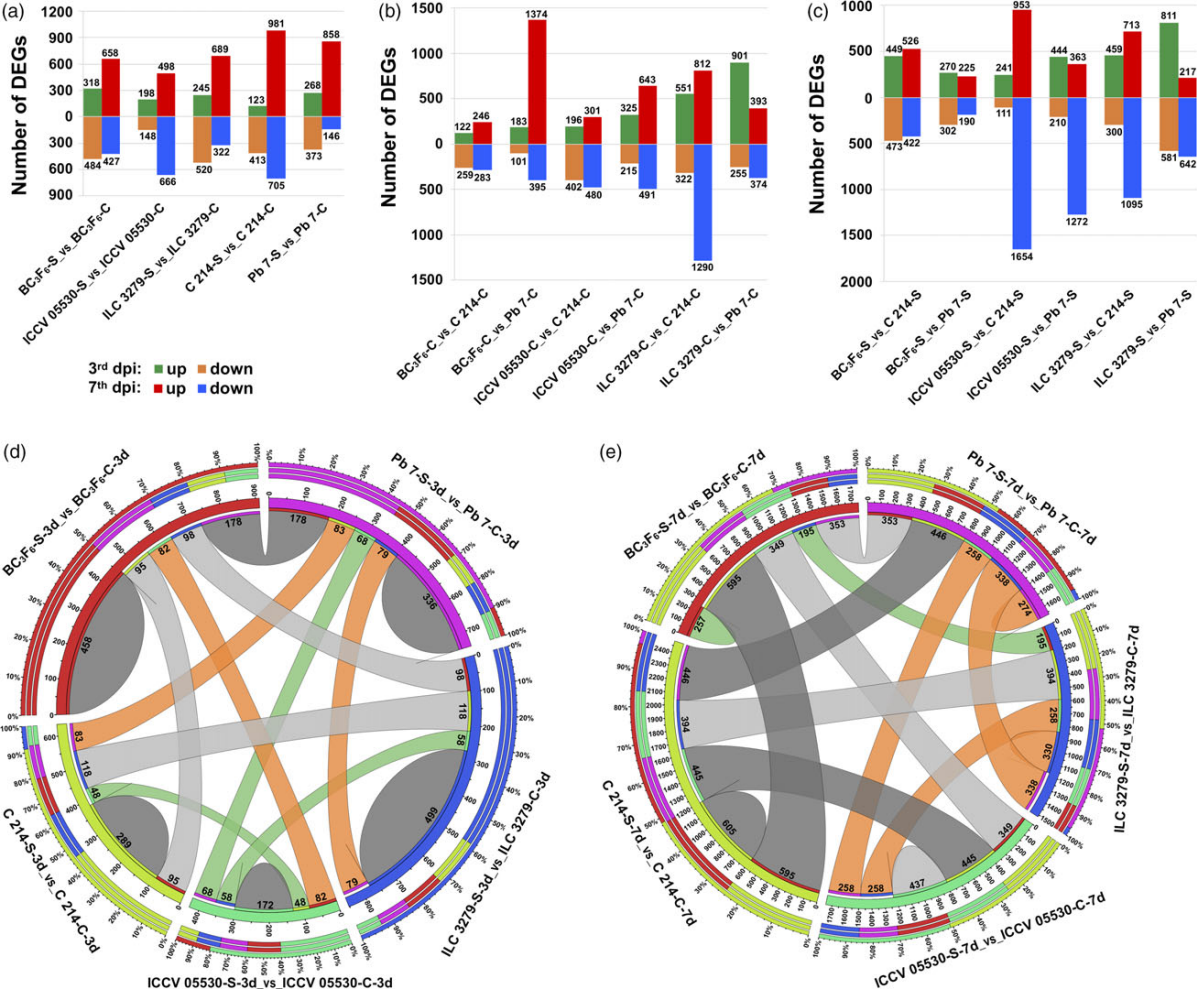
An overview of differentially expressed genes (DEGs) in various combinations of resistant (ICCV 05530, ILC 3279 and BC_3_F_6_) and susceptible (C 214 and Pb 7) genotypes in response to Ascochyta blight (AB) infection. (a) Number of DEGs in each chickpea genotype at 3rd and 7th day post inoculation (dpi) under control and stress conditions are presented in the bar graph. The number of up‐ and down‐ regulated genes are depicted in the form of bars above and below the *x*‐axis, respectively; (b) Number of DEGs between different genotypes at both 3rd and 7th dpi under control conditions; (c) Number of DEGs between different genotypes at both 3rd and 7th dpi under stress conditions. Circos depicting overlapping and specific response of DEGs within AB related genotypes under control and stress conditions at (d) 3rd dpi and (e) 7th dpi. The number of genes showing specific and overlapping response is mentioned.

For studying the differential gene expression, the genes with very low expression values in all the samples were filtered out. A gene was considered to be expressed in a given sample if its FPKM ≥ 1, and the quantification status ‘OK’. Using these criteria**,** a total of 21 527 genes were found to be expressed in at least one of the samples. After filtering the lowly expressed genes, the fold change of each gene was calculated across 34 pairwise combinations using Cuffdiff. In total, 6767 genes were found to show significant differential expression between any two samples. The number of DEGs ranged from 284 (183 up‐regulated; 101 down‐regulated) between BC_3_F_6_‐C‐3d and Pb 7‐C‐3d to 2607 (953 up‐regulated; 1654 down‐regulated) between ICCV 05530‐S‐7d and C 214‐S‐7d (Figure 1a–c). Upon AB infection, the trend of down‐regulation was more intense at 3rd dpi in contrast to 7th dpi where the higher number of genes were found to be up‐regulated in each genotype (Figure 1a). Under control conditions, a significant number of DEGs were identified from different genotype combinations with the highest number of DEGs between ILC 3279 and Pb 7 (901 up‐regulated; 255 down‐regulated) at 3rd dpi and between ILC 3279 and C 214 (812 up‐regulated; 1290 down‐regulated) at 7th dpi (Figure 1b). Similarly, upon infection, the maximum DEGs were found between ILC 3279 and Pb 7 (811 up‐regulated; 581 down‐regulated) at 3rd dpi and between ICCV 05530 and C 214 (953 up‐regulated; 1654 down‐regulated) at 7th dpi (Figure 1c). Further, an overlap between different genotypes under control and stress conditions at both 3rd dpi and 7th dpi was analyzed. It was observed that a large fraction of DEGs was unique to each combination. A total of 1754 and 1903 DEGs exhibited genotype‐specific differential expression patterns under stress at 3rd dpi and 7th dpi, respectively. Under stress, at 3rd dpi, ILC 3279 exhibited highest number of genotype‐specific stress‐responsive DEGs (499) and lowest in ICCV 05530 (172). At 7th dpi, the maximum DEGs were specifically expressed in C 214 (605) and minimum in BC_3_F_6_ line (257) (Figure 1d and e). A total of 1355 genes were differentially expressed at both 3rd and 7th dpi in any of the genotype upon stress. Overall, these results indicate genotype‐ and stage‐ specific response upon fungal infection in chickpea.

**Figure 2:**
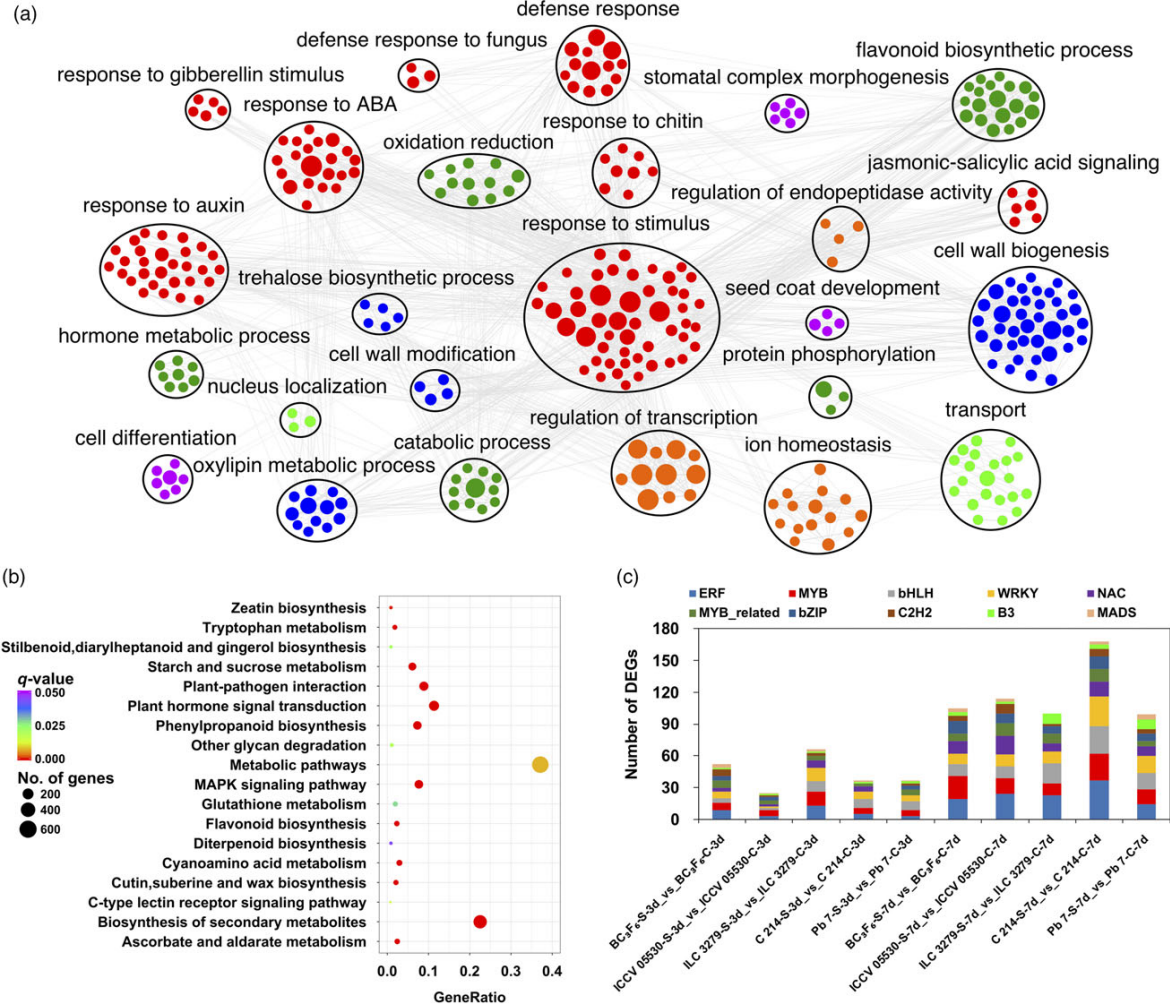
Annotation and pathway analysis of identified differentially expressed genes (DEGs). (a) Gene Ontology enrichment analysis of all identified DEGs; (b) Scatter plot of most enriched KEGG pathways of DEGs. Gene ratio represents the ratio of the number of DEGs in the pathway and total number of DEGs with pathway annotation; *Q* value represents corrected *P*‐value; (c) Number of genes from top 10 TF families showing differential expression patterns in each genotype under control and stress conditions.

Out of 6767 significant DEGs, 5309 were annotated using blastx and the gene ontology (GO) terms were assigned to 4559 genes. For these 4559 genes, a total of 10 962 GO terms were obtained. It was seen that the GO terms for DEGs were uniformly assigned to each of the biological process (3632), molecular function (3744) and cellular component (3586) categories. GO enrichment analysis of DEGs revealed that under biological process category, oxidation‐reduction process (GO:0055114), regulation of transcription (GO:0006355), defense response to fungus (GO:0050832), response to chitin (GO:0010200), response to salicylic acid (GO:0009751), response to jasmonic acid (GO:0009753), response to abscisic acid (GO:0009737), ethylene‐activated signaling pathway (GO:0009873) and response to auxin (GO:0009733) were the most significantly enriched terms. The significantly enriched GO terms were further clustered to obtain the highly interconnected GO clusters (Figure 2a). Among molecular function category, ATP binding (GO:0005524), protein serine/threonine kinase activity (GO:0004674), oxidoreductase activity (GO:0016491), peroxidase activity (GO:0004601), hydrolase (GO:0016787) and monooxygenase activity (GO:0004497) were significantly over‐represented. In cellular component category, plant‐type cell wall (GO:0009505), apoplast (GO:0048046) and anchored component of membrane (GO:0031225) were the most enriched ones. Further, the pathway analysis of DEGs was carried out to understand their molecular mechanisms using KEGG database. The DEGs were found to represent a total of 134 pathways. The enrichment analysis suggested that under AB infection, metabolic pathways (ko01100), biosynthesis of secondary metabolites (ko01110), plant hormone signal transduction (ko04075) and plant‐pathogen interaction (ko04626) were among the most enriched pathways (Figure 2b). In the study, 891 transcription factor (TF) encoding genes belonging to 50 families were differentially expressed. Among these, bHLH (91), ERF (90), MYB (75), NAC (68) and WRKY (65) were the most over‐represented TF families. A significant number of other TF families like C2H2, bZIP and MADS were also found to show different expression patterns under stress conditions. The differential expression specificity of 10 most abundant TF families in all genotypes under stress condition was studied (Figure 2c). It was seen that at 7th dpi, the number of differentially expressed TF encoding genes were higher in all genotypes. A very limited number of TF families showed preferential differential expression pattern in different genotypes under stress. For instance, the number of differentially expressed WRKY members were relatively more in susceptible genotypes as compared to resistant ones.

**Figure 3:**
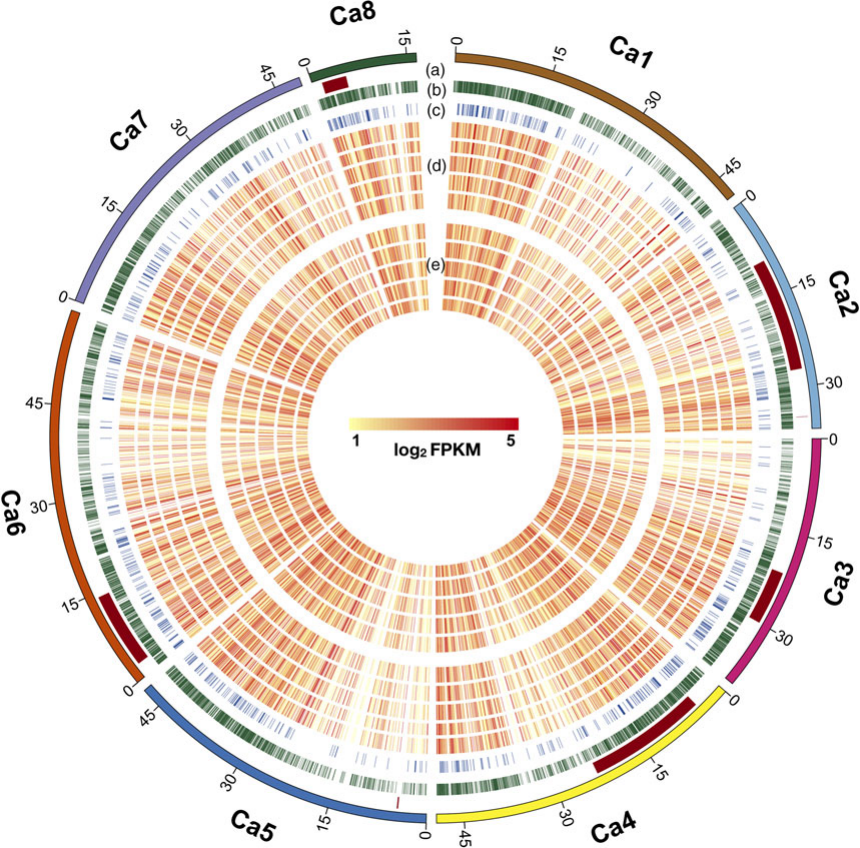
A circular plot showing genome‐wide distribution of Ascochyta blight (AB) resistance QTLs, differentially expressed genes (DEGs), transcription factors (TFs) and expression of genes at 3rd and 7th day post inoculation (dpi). Five different tracks (out to in) of the circular plot shows following: (a) AB resistance QTLs; (b) DEGs; (c) Differentially expressed TFs; (d) Expression of genes (log_2_ FPKM) in different genotypes at 3rd dpi (moving from out to in; ICCV 05530, ILC 3279, BC_3_F_6_, C 214 and Pb 7); (e) Expression of genes (log_2_ FPKM) in different genotypes at 7th dpi (moving from out to in; ICCV 05530, ILC 3279, BC_3_F_6_, C 214 and Pb 7).

For AB resistance, several QTLs have been reported until now (see Sagi *et al*., 2017). These QTLs were physically mapped on six chickpea pseudomolecules by using the markers flanking these QTLs. Out of the 6767 DEGs, 1138 co‐localized with 11 AB resistance QTLs. Among these co‐localized genes, 226 DEGs were novel. Maximum DEGs were located in QTLs reported by Sabbavarapu *et al*. (2013) which included QTL *AB‐Q‐SR‐4‐1* (176) present on pseudomolecule Ca4, *AB‐Q‐APR‐6‐1* (227) and *AB‐Q‐APR‐6‐2* (37) located on Ca6. The genomic localization of AB resistance QTLs, DEGs, TFs and an overall expression of the DEGs in each genotype at both time points are illustrated in Figure 3.

#### Expression trends across the chickpea genotypes under control and stress conditions

**Figure 4:**
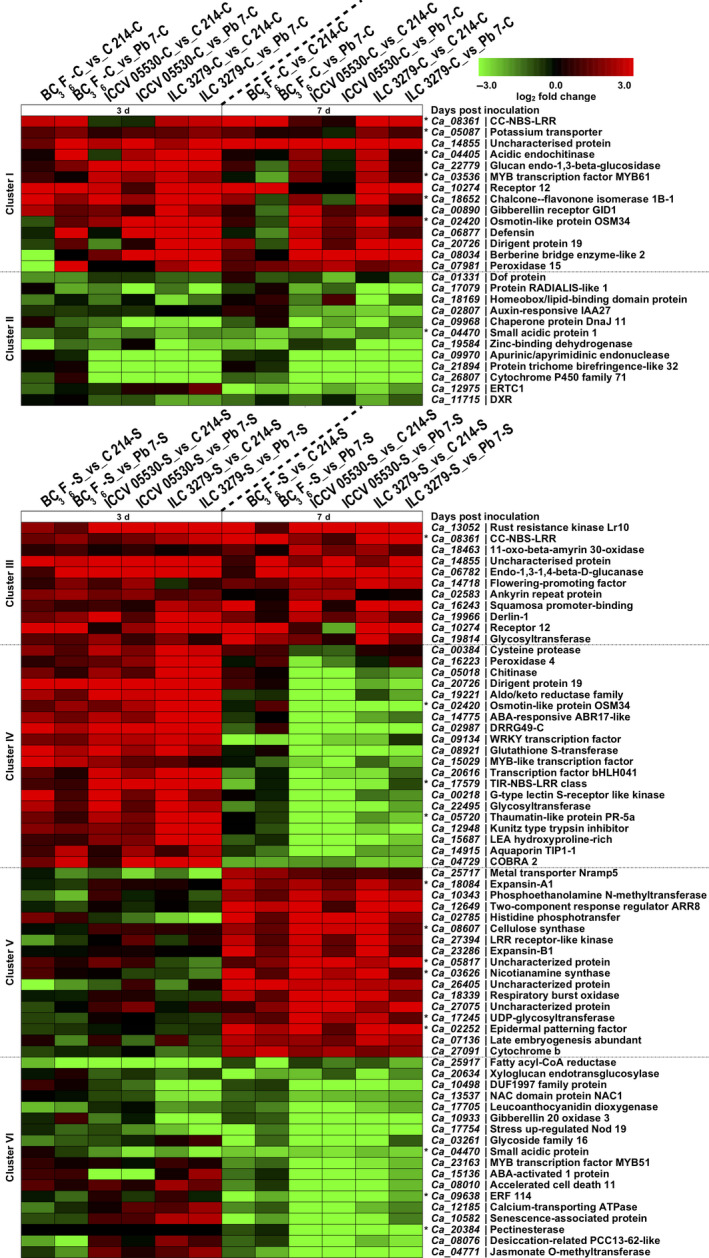
Clustering of expression profiles of differentially expressed genes (DEGs) under control and stress conditions. The clustering was performed on log_2_ fold change for each gene under different combinations. The genes showing similar expression trend have been grouped together into six clusters (Cluster I to Cluster VI) and selected representative genes from each cluster are depicted in the form of heat maps. Cluster I and II show the differential expression trend of genes between resistant (ICCV 05530, ILC 3279 and BC_3_F_6_) and susceptible (C 214 and Pb 7) genotypes under control conditions. Cluster III to VI shows the differential expression trend of genes between resistant and susceptible genotypes under stress conditions. The asterisk (*) shows that the gene is present in one of the previously reported Ascochyta blight (AB) QTLs. The color scale at the top shows log_2_ fold change.

Defense response involves substantial transcriptional reprogramming in plants. Using clustering analysis, we identified genes with similar expression trends across resistant (ILC 3279, ICCV 05530 and BC_3_F_6_) and susceptible genotypes (C 214 and Pb 7) at different time points. The DEGs were clustered into six major clusters on the basis of similar expression profiles (Figure 4). Under control conditions, a trend of up‐regulation was more profound in resistant genotypes. The Cluster I consisted of genes showing induced expression in resistant genotypes at either 3rd dpi or 7th dpi. The GO enrichment of these genes revealed that most of these genes were involved in response to salicylic acid, metabolic process, oxidation‐reduction process, flavonoid biosynthetic process and response to biotic stimulus. Interestingly, disease resistance genes like chitinases (*Ca_04405*), CC‐NBS‐LRR (*Ca_08361*) and dirigent protein (*Ca_20726*) were significant members of this cluster (Figure 4). Under control conditions, another cluster (Cluster II) was identified which showed repressed expression of genes in resistant genotypes at either 3rd or 7th dpi. These genes were mainly associated with response to auxin, catabolic process, oxidoreductase activity, heme binding and xylan acetylation. Auxin responsive elements, homeobox related and TFs like ERF (*Ca_12975*) and Dof (*Ca_01331*) were included in this cluster (Figure 4). Further, when chickpea plants were exposed to AB stress, a cluster (Cluster III) showing induced expression at both 3rd and 7th dpi in resistant genotypes was observed. This cluster mainly contained genes involved in pathogen recognition and defense response like leucine‐rich receptor‐like kinase, NBS‐LRR proteins, oxidation‐reduction process, cuticle development and fatty acid biosynthesis. Cluster IV included genes showing significant up‐regulation in resistant genotypes at 3rd dpi. It contained genes involved in defense response to fungus, chitin catabolic process, signal transduction, peroxidase activity and oxidation‐reduction process. A number of stress‐responsive genes like pathogenesis‐related (PR) proteins, chitinases, Kunitz‐type trypsin inhibitor, glutathione‐S‐transferase (GST), TIR‐NBS‐LRR, and dirigent proteins along with TFs like bHLH, WRKY and MYB were a major part of this cluster. It was observed that a gene coding for disease resistance response protein, DRRG49‐C (*Ca_02987*) exhibited an average log_2_ fold change of 5.5 across resistant genotypes compared to the susceptible genotypes. A significant number of the genes of this cluster were found to be considerably repressed at 7th dpi in resistant genotypes. These included several stress‐responsive genes like dirigent, syntaxin, COBRA, Kunitz‐type trypsin inhibitor and aquaporins. Cluster V consisted of genes showing up‐regulation at only 7th dpi in resistant genotypes. This cluster included genes related to cell wall biogenesis like cellulose synthase (*Ca_08607*), cell wall loosening like expansin and ion homeostasis including Nramp5 (*Ca_25717*). Cluster VI exhibited induced expression in susceptible genotypes at either 3rd or 7th dpi. The genes of this cluster were mainly involved in defense response, proteolysis, response to abscisic acid, response to salicylic acid, hydrolase activity, jasmonic acid and ethylene‐mediated signaling pathway, protein phosphorylation and leaf senescence. Genes like senescence‐associated protein (*Ca_10582*), accelerated cell death 11 (*Ca_08010*), desiccation‐related protein (*Ca_08076*), jasmonate O‐methyltransferase (*Ca_04771*), leucoanthocyanidin dioxygenase‐like protein (*Ca_17705*), pectinesterase (*Ca_20384*) and calcium‐transporting ATPase (*Ca_12185*) and TFs encoding for NAC, MYB and ERF were up‐regulated in susceptible genotypes (Figure 4). Interestingly, majority of the genes of this cluster like senescence‐associated and accelerated cell death proteins were repressed at 3rd dpi and induced at 7th dpi in susceptible genotypes.

### High‐throughput small RNA sequencing

For the identification of AB stress‐related miRNAs in chickpea, a total of 20 small RNA libraries were constructed and sequenced. A total of 532.8 million reads with an average of 26 million reads per sample were generated. After subsequent steps of filtering low quality reads and trimming, a set of 517.2 million high quality reads was retained for further analysis. Around 60 million reads with length <18 nt or >35 nt were discarded, followed by removal of reads mapping to repeats, ribosomal RNA (rRNA), transfer RNA (tRNA) and small nucleolar RNA (snoRNA) (Table 1). The length distribution of the unique small RNA reads indicated that 24 nt (51.2%) small RNAs were the most abundant class followed by 21 nt (7.9%), 23 nt (7.7%) and 22 nt small RNAs (6.1%) (Figure S1). The high representation of 24 nt small RNAs implies the abundant representation of endogenous siRNAs in all the samples analyzed, in concurrence with previous studies (Candar‐Cakir *et al*., 2016; Jain *et al*., 2014).

#### Identification of known and novel miRNAs

In order to identify conserved/known miRNAs in chickpea, the filtered reads were searched against the miRNAs of other plant species from miRBase. A total of 11.2 million reads were mapped to miRbase which resulted in the identification of 478 unique conserved miRNAs from all samples (Table S1). Along with conserved miRNAs, plants also harbor novel miRNAs that are species‐ or lineage‐ specific. Therefore, the reads which could not map to miRBase were subjected to novel miRNA prediction. A total of 4.2 million reads which mapped onto chickpea genome with no mismatch were subjected to processing by miRDeep‐P. In brief, the mapped reads were extended to obtain precursor sequences which were further folded into potential stem‐loop structures using Vienna package. The characteristic miRNA precursor secondary structures were filtered and processed. After the removal of miRNAs which do not meet the plant miRNA criteria and were represented by less than 10 reads in a sample, a final set of 173 unique novel miRNAs was obtained (Table S1).

The total number of miRNAs ranged from 339 in Pb 7‐C‐7d to 471 in BC_3_F_6_‐S‐3d. The length of identified miRNAs ranged from 20–24 nt, with 21 and 24 being the most abundant (Table S1). The 20–24 nt length of miRNAs is in agreement with the products of DCL cleavage activity (Reinhart *et al*., 2002). The nucleotide composition of the mature miRNA candidates was evaluated and it was seen that majority of 21 nt long miRNAs were characterized by a 5′‐uridine residue. The abundance of 5′‐uridine residue in 21 nt length miRNAs has been observed in many plant species and is a characteristic attribute of DCL1 cleavage and AGO1 association (Reinhart *et al*., 2002; Rajagopalan *et al*., 2006; Figure S2). The average GC content of chickpea miRNAs was found to be about 48% (Table S1). The GC content of chickpea miRNAs was similar to the GC content of *Medicago* (44%), *Arabidopsis* (45%), soybean (46%) and grapevine (50%). The similarity‐based clustering of identified miRNAs grouped them into 240 families. Among 240 families, miR166 was the largest family with 46 members followed by miR156 with 44 and miR171 with 33 members. Furthermore, five novel miRNAs were found to be clustered with conserved miRNAs (Table S1). These miRNAs might constitute the recently evolved members of conserved miRNA families.

#### Differentially expressed miRNAs during AB infection

**Figure 5:**
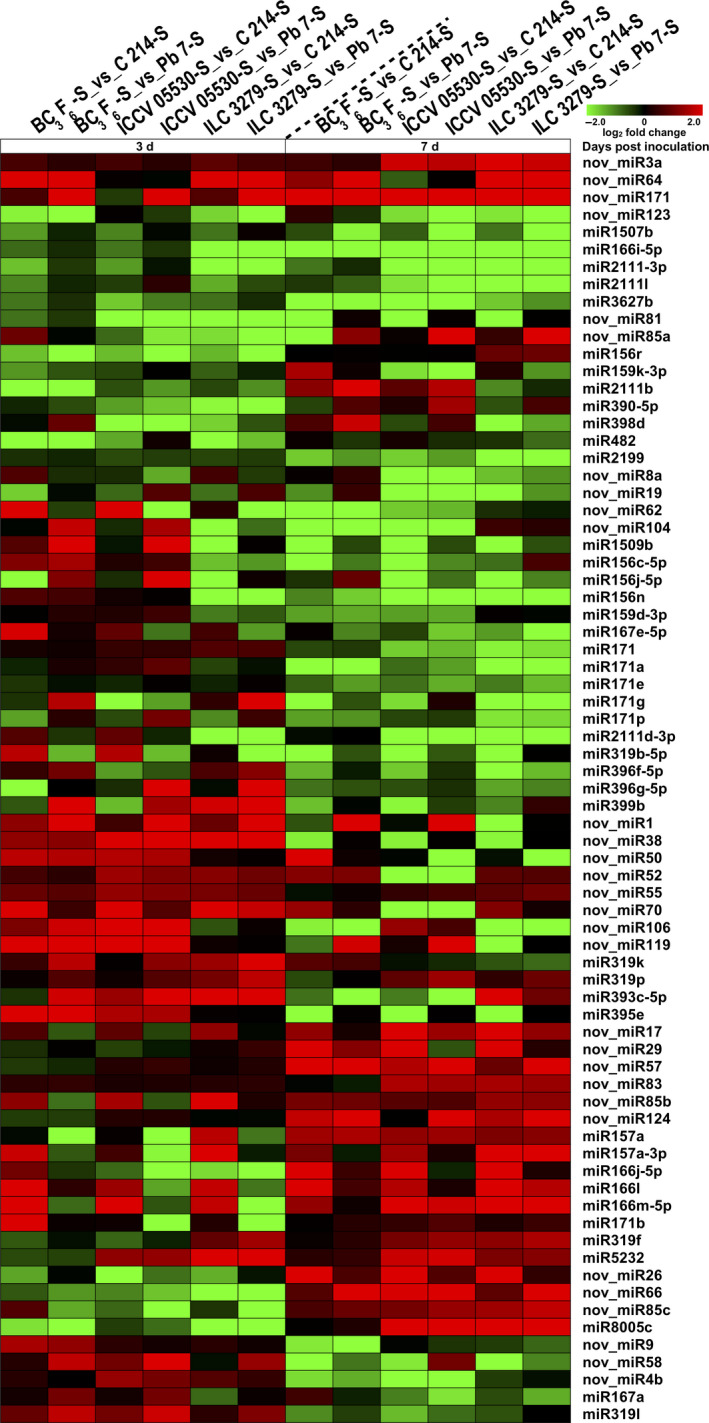
Differential expression of stress‐responsive conserved and novel miRNAs in Ascochyta blight resistant (ICCV 05530, ILC 3279 and BC_3_F_6)_ and susceptible (C 214 and Pb 7) genotypes are shown as a heatmap. The color scale at the top shows log_2_ fold change.

To distinguish miRNAs that respond to AB, the identified miRNAs were studied for their expression patterns in all the libraries. The majority of the identified miRNAs were expressed in more than one sample. In the present study, 297 miRNAs showed significant differential expression patterns across different combinations. The majority of miRNAs showed a trend of down‐regulation under stress conditions. A significant number of miRNAs were differentially expressed in a genotype‐ and stage‐ specific manner. Under stress, around 25% of the differentially expressed miRNAs were genotype‐specific at 3rd dpi and nearly 44% miRNAs at 7th dpi. For example, miR156h‐3p was highly induced (average log_2_ fold change 4) at both 3rd and 7th dpi specifically in ILC 3279 genotype. Further, to interrogate the miRNAs involved in AB resistance, we looked into miRNAs that exhibited similar expression patterns in all resistant genotypes including introgression line by comparing resistant and susceptible genotypes under stress (Figure 5). In total, three novel miRNAs (nov_miR3a, nov_miR64, nov_miR171) showed up‐regulation at both the 3rd and 7th dpi in resistant genotypes. A total of seven miRNAs including miR3627b, miR2111l, miR2111‐3p, miR1507b, nov_miR123, miR166i‐5p and nov_miR81 were down‐regulated in resistant genotypes at both 3rd and 7th dpi. A trend of down‐regulation of miRNAs was more profound at 7th dpi. A total of 8 and 21 miRNAs were specifically down‐regulated at 3rd and 7th dpi, respectively, in resistant genotypes. Similarly, a set of 12 and 14 miRNAs were up‐regulated in resistant genotypes at 3rd and 7th dpi, respectively. Interestingly, few miRNAs like nov_miR85c, nov_miR66 and miR8005c were down‐regulated at 3rd dpi and up‐regulated at 7th dpi in resistant genotypes. In contrast, nov_miR4b, nov_miR58, nov_miR9, miR319l and miR167a were up‐regulated at 3rd dpi and down‐regulated at 7th dpi in resistant genotypes (Figure 5). A significant fraction of miRNAs showed similar trends in both resistant genotypes (ILC 3279 and ICCV 05530) but contrast expression pattern in introgression line. These miRNAs mainly included isoforms of miR398, miR390, miR4414 and many novel miRNAs like nov_miR126 and nov_miR131a.

### Target prediction via *in silico* and degradome approaches

Plant miRNAs usually have perfect or near‐perfect complementarity with their targets allowing the identification of targets using *in silico* tools. Using psRNATarget server, we could identify 1735 targets for a total of 593 (429 known and 164 novel) miRNAs. Further, using degradome sequencing, a total of 145.8 million sequence reads were generated with an average of 14.6 million reads per sample (Table S2). After consecutive steps of filtering, 134.4 million reads were obtained which were processed for identification of cleavage sites. For clarity, the samples have been designated as genotype‐condition. For instance, ILC 3279‐C represents genotype ILC 3279 under control conditions. An average of 179 non‐redundant targets with *P*‐value ≤ 0.05 and category ≤ 4 in each sample were identified. The maximum targets were identified for ILC 3279‐C (201) and minimum for Pb 7‐C (151). For each sample, the maximum cleavage sites belonged to category 0 (average 41.1%) and minimum to category 4 (average 12.7%) (Table S2). These cleavage sites were represented in the form of target plots (T‐plots). Using degradome sequencing, we could identify 552 targets for a total of 407 (324 known and 83 novel) miRNAs.

Through *in silico* and degradome analysis, a total of 2131 targets were identified for almost all (96.6%) miRNAs (Table S1). It was seen that the maximum targets were obtained for members of miR396 family (148), followed by miR167 (106) and miR156 (80). The annotation of the targets revealed that they mainly belong to TFs, phytohormones, stress‐responsive genes, disease resistance genes like NBS‐LRR, cellular enzymes like kinases, reductases and phosphatases. TF encoding mRNA transcripts also represented a considerable fraction of miRNA targets. In total, 192 TFs belonging to 35 families were identified as targets. The maximum targets were from MYB family (17.2%), followed by bHLH (7.8%) and ARF (6.8%). GO enrichment analysis was further performed to elucidate the potential role of miRNA targets in response to AB stress in chickpea. The targets were uniformly assigned to 1470 biological processes, 1477 cellular components and 1503 molecular functions. Among the most enriched biological processes were RNA modification (GO:0009451), plant‐type secondary cell wall biogenesis (GO:0009834), defense response (GO:0006952), response to jasmonic acid (GO:0009753), signal transduction (GO:0007165) and response to salicylic acid (GO:0009751). Among molecular functions, the most significant GO terms were endonuclease activity (GO:0004519), miRNA binding (GO:0035198), oxidoreductase activity (GO:0016722) and xylan O‐acetyltransferase activity (GO:1990538). In cellular component category, nucleus (GO:0005634), plasma membrane (GO:0005886) and mitochondrial inner membrane pre‐sequence translocase complex (GO:0005744) were the most over‐represented terms (Figure S3).

**Figure 6:**
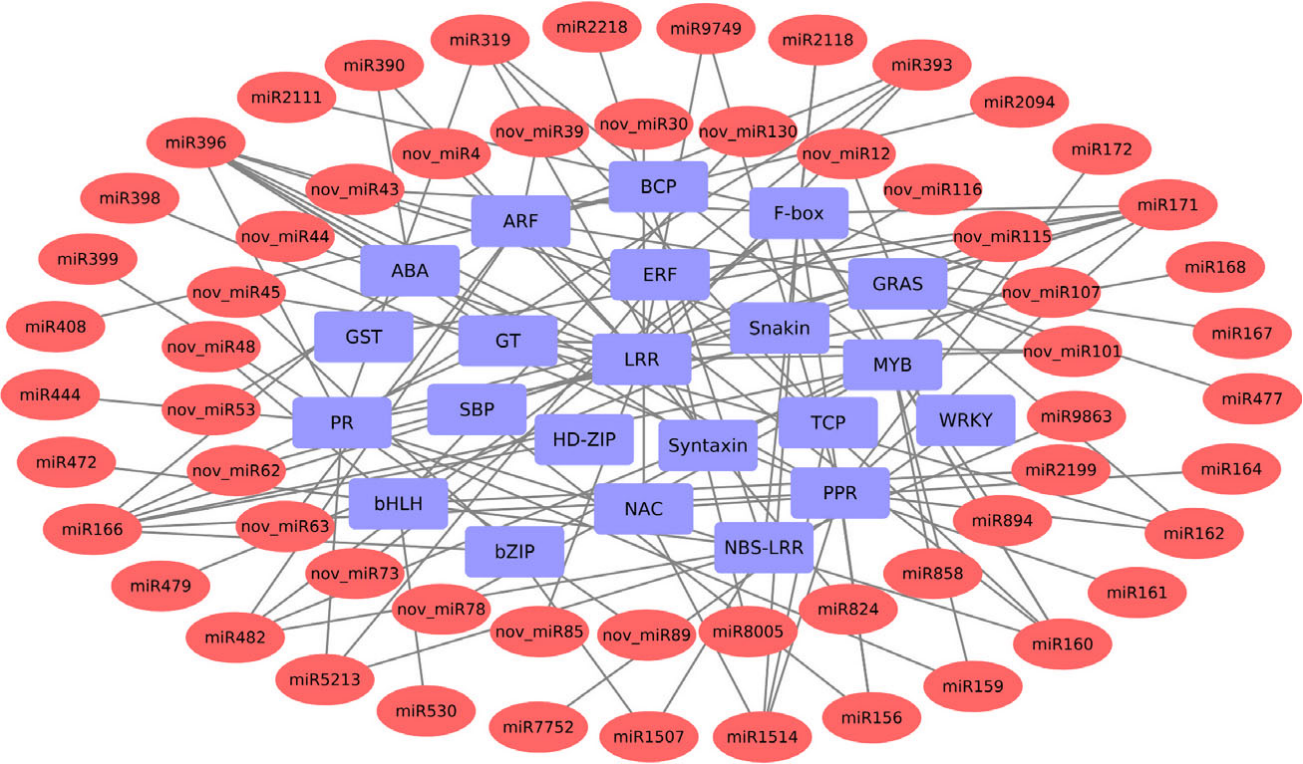
A network representing the relationships between miRNAs and their target genes associated with Ascochyta blight response. The red color eclipses represent the miRNAs and blue colored rectangles represent the target genes. ARF, auxin response factor; BCP, blue copper protein; GT, glycosyltransferase; NBS‐LRR, nucleotide‐binding site leucine‐rich repeat; PPR, pentatricopeptide repeat protein; PR, pathogenesis‐related proteins; and SBP, SQUAMOSA promoter binding protein.

Further, KEGG annotation was carried out to explore the pathways in which the identified miRNA targets are involved. A total of 214 pathways were identified indicating the highly diverse functions of these targets. The most enriched pathways were the ones related to stress response including plant‐pathogen interaction, plant hormone signal transduction, biosynthesis of secondary metabolites and MAPK signaling pathway (Figure S4). Further, to investigate the association of AB responsive miRNAs with their targets, network analysis was performed using Cytoscape platform. For network construction, stress‐responsive miRNAs and genes involved in AB stress response were included. These stress‐responsive genes included genes encoding for PR proteins, glycosyltransferases (GT), Pentatricopeptide repeat proteins (PPR) and disease resistance genes like NBS‐LRR and Snakin. The network also included several TFs like NAC, ERF and ARF (Figure 6).

### Correlation analysis of miRNAs expression profiles and their target genes

**Figure 7:**
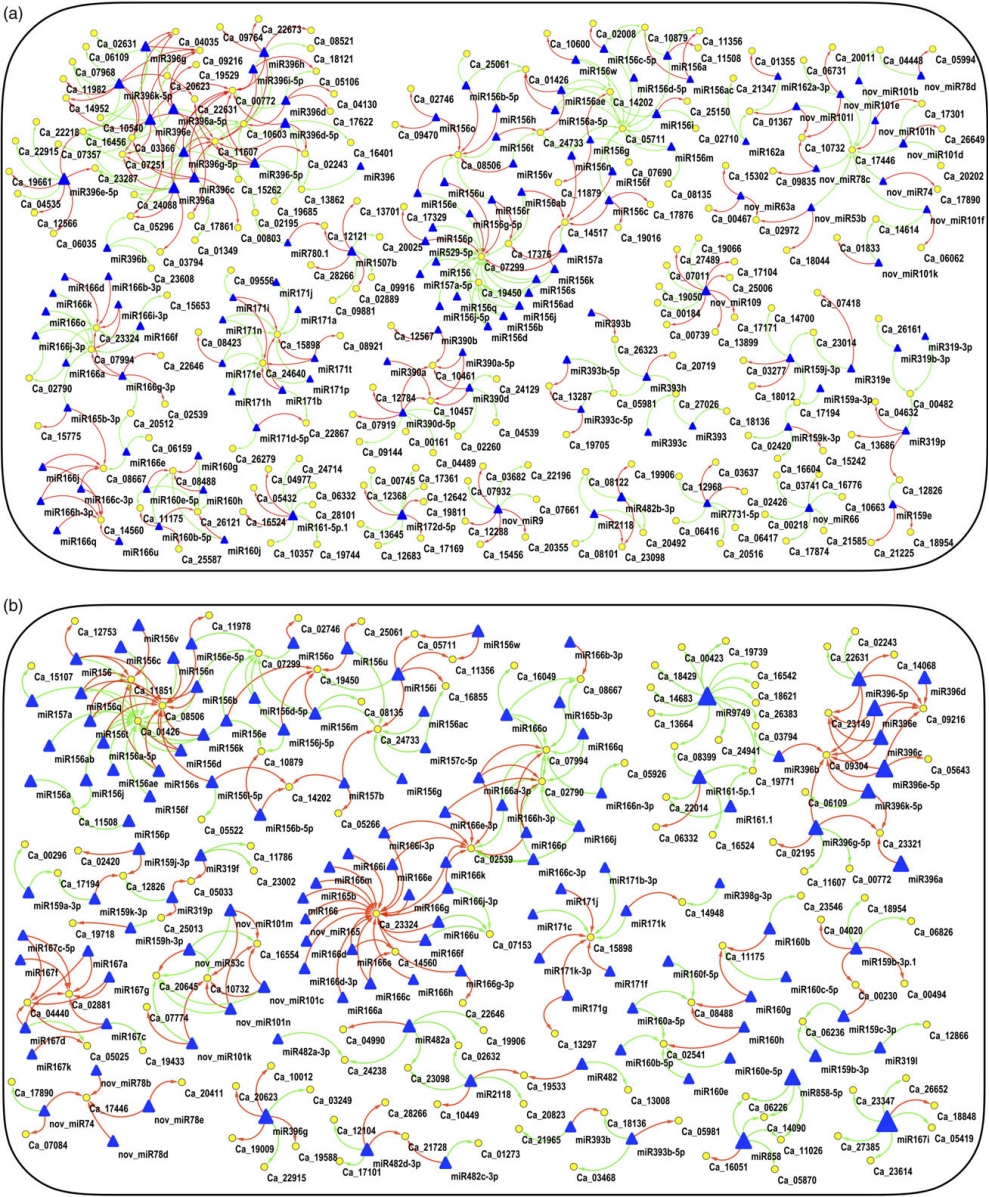
Integrated correlation networks of the identified miRNAs and their target genes in resistant (ICCV 05530, ILC 3279 and BC_3_F_6_) and susceptible (C 214 and Pb 7) genotypes. The sub‐networks with ≥ five edges are shown. Triangles represent miRNAs; ellipses represent the target gene; red edges/lines represent positive correlation and green represents negative correlation (a) at 3rd day post inoculation (dpi); (b) 7th dpi.

Further, the expression of both AB responsive miRNAs (from small RNA‐seq) and their target genes (from RNA‐seq) were integrated to infer the mediatory role of miRNAs during AB infection. The correlation analysis of miRNA and their target mRNA expression profiles using Pearson correlation coefficient identified a total of 757 and 693 miRNA‐mRNA interaction pairs across all combinations under stress conditions at 3rd and 7th dpi, respectively. These interactions can be either coherent or non‐coherent. Coherent interactions are the ones in which the expression of target mRNA is more when the expression of miRNA is less and vice versa. In contrast, non‐coherent are the ones in which both miRNA and its target mRNA have the similar expression (Shkumatava *et al*., 2009). Out of 757 pairs, 371 were non‐coherent (positive correlation) and 386 pairs were coherent (negative correlation) at 3rd dpi (Figure 7a). Similarly, at 7th dpi, out of total 693 pairs, 357 were non‐coherent and 336 pairs were coherent (Figure 7b). We further analyzed coherent interactions in detail. At 3rd dpi, 386 coherent pairs consisting of 274 genes and 247 miRNAs were found. Similarly, at 7th dpi, 336 coherent pairs comprising of 262 genes and 232 miRNAs were identified. The higher number of genes as compared to the miRNAs targeting them support the findings that a single miRNA has the capability to cleave multiple targets.

**Figure 8:**
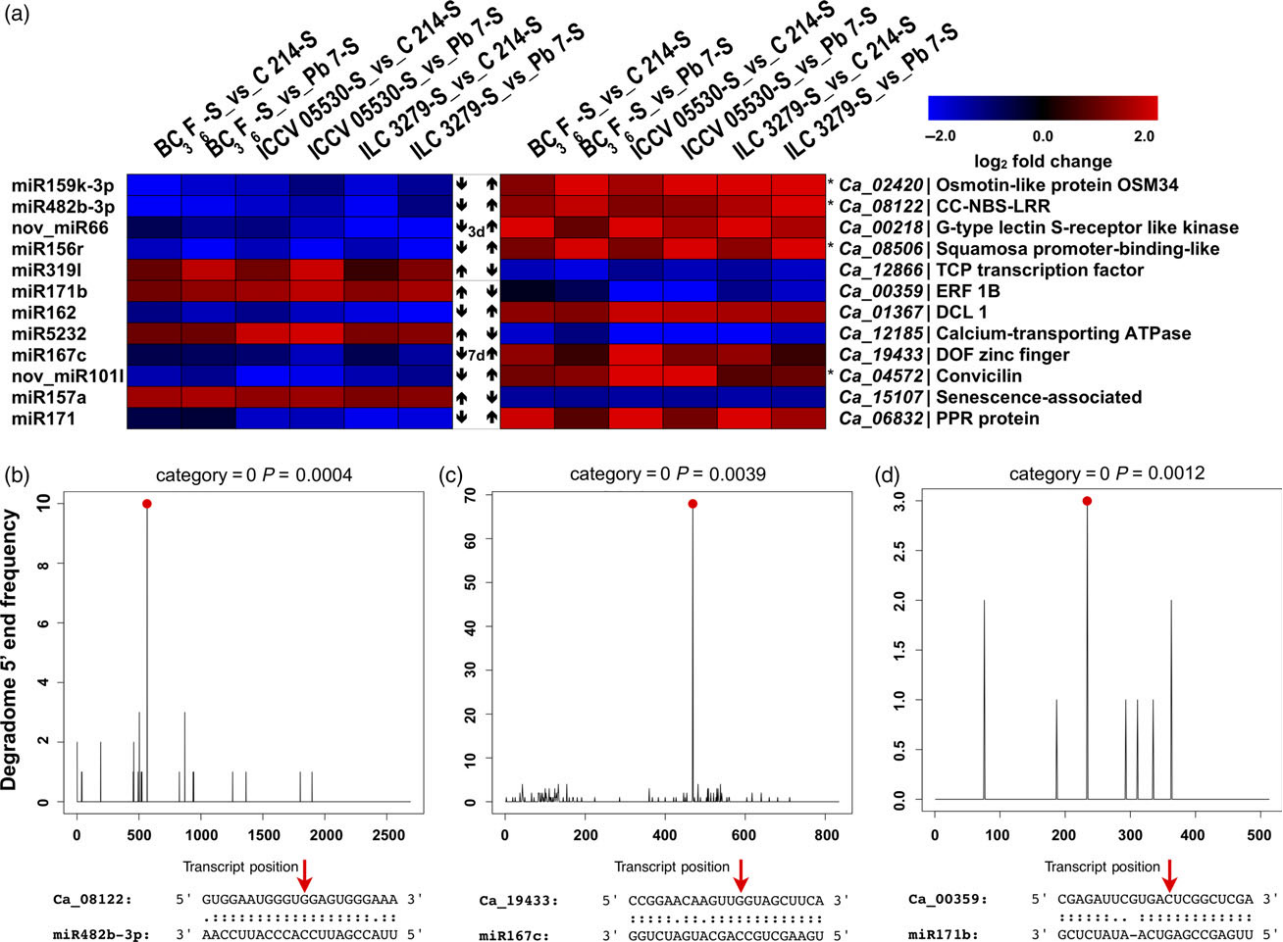
Expression profiles of important miRNA‐mRNA interaction pairs and their validation. (a) A combined view of the expression level of selected coherent pairs of differentially expressed miRNAs and their target genes in chickpea genotypes upon Ascochyta blight (AB) infection. The heat map on left indicates the miRNA expression and the heat map in right represent the corresponding target expression between resistant (ICCV 05530, ILC 3279 and BC_3_F_6_) and susceptible (C 214 and Pb 7) genotypes. The asterisk (*) shows that the gene is present in one of the previously reported AB resistance QTLs. T‐plots and miRNA‐mRNA alignments validated by degradome sequencing where (b) miR482b‐3p cleaves NBS‐LRR (*Ca_08122*) gene; (c) miR167c cleaves Dof zinc finger (*Ca_19433*) gene; (d) miR171b cleaves ERF (*Ca_00359*) gene. The red dots and arrows represent the cleavage nucleotide positions on the target genes.

From these coherent pairs, we could identify 12 pairs that exhibited contrasting expressions of both mRNA and miRNA across resistant (including BC_3_F_6_ line) and susceptible genotypes (Figure 8a). From these pairs, five and seven pairs were identified from 3rd and 7th dpi, respectively. It was seen that a higher number of miRNAs were down‐regulated and their corresponding targets were up‐regulated in resistant genotypes when compared with the susceptible genotypes. In all resistant genotypes, at 3rd dpi, three miRNAs, namely, miR159k‐3p, miR482b‐3p and miR156r were down‐regulated and their targets osmotin‐like protein, NBS‐LRR and Squamosa promoter binding like protein, respectively, were up‐regulated. Along with these known miRNAs, a novel miRNA, nov_miR66 targeting a G‐type lectin S‐receptor like Serine threonine kinase was also seen to exhibit the similar trend. In contrast, miR319l was up‐regulated and its target, a TCP transcription factor was down‐regulated in all resistant genotypes. At 7th dpi, three miRNAs including miR171b, miR5232 and miR157a were up‐regulated and their targets, an ERF gene, calcium‐transporting ATPase and senescence‐associated protein were down‐regulated in resistant genotypes. In contrast, four miRNAs, namely, miR162, miR167c, nov_miR101l and miR171 were down‐regulated and their targets, a Dicer like gene, Dof zinc finger, convicilin and a PPR protein were up‐regulated in resistant genotypes. Interestingly, at both the time points the disease resistance genes, such as, NBS‐LRR (3rd dpi), osmotin (3rd dpi) and PPR (7th dpi) protein were up‐regulated and the miRNAs targeting them were down‐regulated in resistant genotypes as compared to the susceptible genotypes (Figure 8a).

Out of these 12 miRNA‐mRNA pairs, five pairs were also validated by degradome sequencing. These pairs include miR482b‐3p: CC‐NBS‐LRR (*Ca_08122*), miR167c: Dof zinc finger protein (*Ca_19433*), miR171b: ERF (*Ca_00359*), miR157a: senescence‐associated protein (*Ca_15107*) and miR5232: calcium‐transporting ATPase (*Ca_12185*) (Figure 8b–d, Figure S5a and b). Four genes from these 12 pairs were localized in previously reported AB resistance QTLs. *Ca_02420* was co‐localized with QTL on Ca8 by Anbessa *et al*. (2009), *Ca_08122* on Ca3 by Tar'an *et al*. (2007), *Ca_08506* on Ca6 and *Ca_04572* on Ca4 by Sabbavarapu *et al*. (2013).

### Validation of differential gene and miRNA expression

The quantitative reverse transcription‐polymerase chain reaction (qRT‐PCR) analysis was carried out to validate the expression patterns of genes and miRNAs obtained from RNA and small RNA sequencing. The expression of nine randomly selected genes and seven miRNAs was validated via qRT‐PCR in all 20 samples. The primer sequences used for both genes and miRNA validation are presented in Table S3. Similar expression trends of genes and miRNAs (up‐regulation or down‐regulation) were observed in qRT‐PCR analysis as that of high‐throughput sequencing for most of the samples. The means of the correlation coefficients of the qRT‐PCR experiments, and the sequencing results for the genes and miRNAs were 0.78 and 0.73, respectively. For individual genes, the correlation value varied from 0.71 to 0.85 and for miRNAs, it ranged from 0.70 to 0.77 (Figure S6). These results suggest a fine agreement between the results obtained through high‐throughput sequencing and qRT‐PCR.

## Discussion

**Figure 9:**
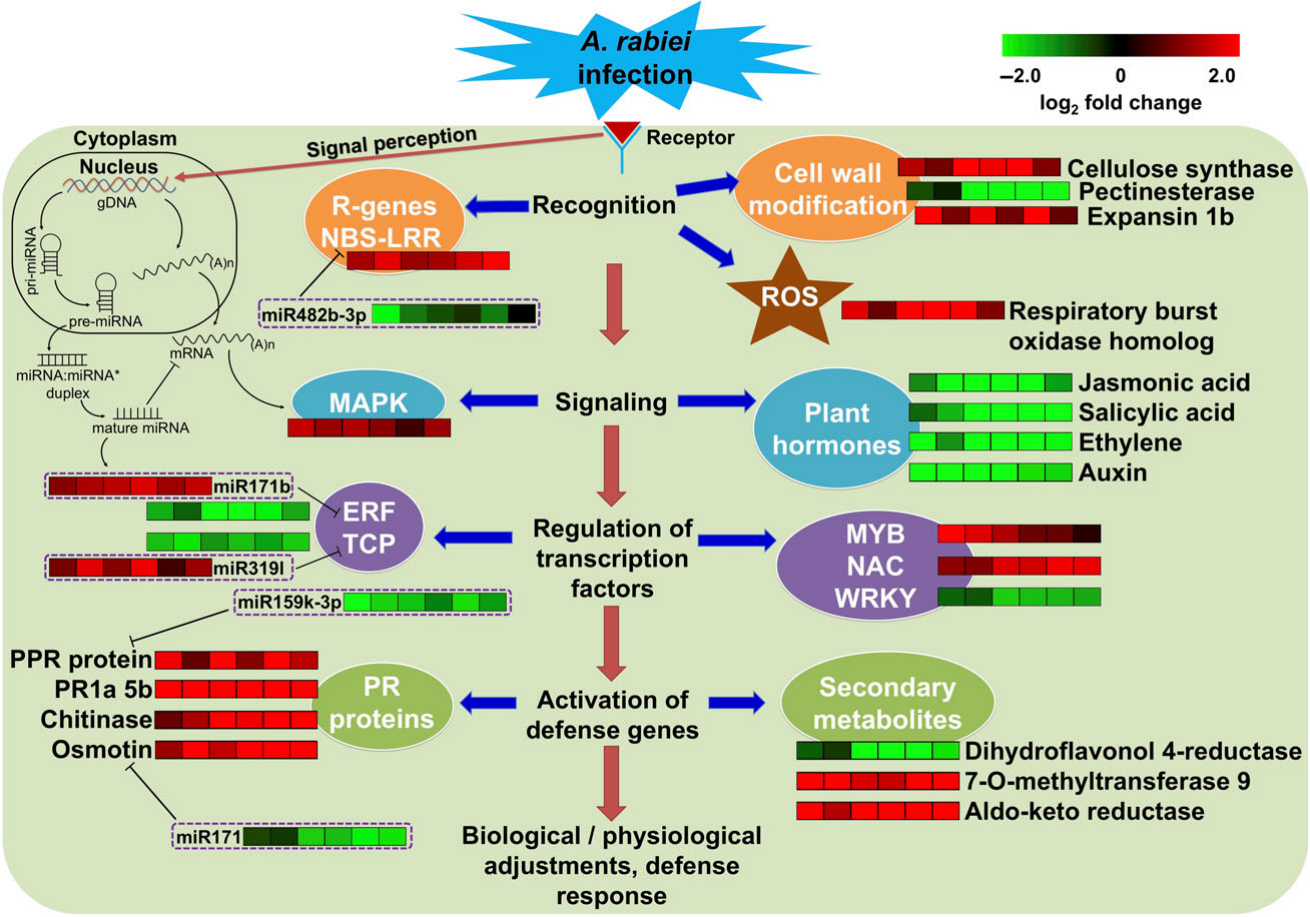
A hypothetical scheme showing summary of the cascades of various physiological and biochemical events incurred during the interaction of *A. rabiei* with chickpea in resistant genotypes. The heat maps indicate the up‐regulation (red) and down‐regulation (green) of gene/miRNA in resistant genotypes when compared with susceptible genotypes under stress conditions.

The availability of diverse germplasm and high‐throughput sequencing technologies provide a distinguished opportunity to understand the molecular basis of variability in stress response. In this study, three high‐throughput approaches, namely, transcriptomics, small RNA and degradome sequencing were used for better understanding of genetic and molecular mechanisms behind AB stress resistance in chickpea. We investigated four well‐characterized chickpea genotypes (ILC 3279, ICCV 05530, Pb 7 and C 214) and an introgression line (BC_3_F_6_) for their response to AB by combining the analyses from all the three approaches. Understanding the molecular basis of AB resistance can accelerate the development of stress‐resistant varieties in chickpea through both molecular breeding and genetic engineering approaches. Although, a few genes involved in AB response in chickpea have been reported (Coram and Pang, 2006; Leo *et al*., 2016), the molecular mechanism underlying AB resistance remains largely unknown. To understand the molecular response of plants to adapt to AB stress, we performed a comprehensive transcriptome analysis of the AB resistant and susceptible chickpea genotypes. The reference guided assembly generated a total of 31 459 genes, a number higher than the number of genes identified based on genome assembly (Varshney *et al*., 2013). This study identified 3190 novel genes, thus exhibiting the potential of RNA‐seq in the discovery of novel genes in the sequenced genomes as well. Similar results were also observed in other studies in chickpea (Garg *et al*., 2016; Kudapa *et al*., 2018). A total of 6767 genes showed differential expression pattern in different genotypes under different conditions at different time points. The differential expression pattern of genes in resistant and susceptible genotypes and various pathways involved enhanced our understanding about the AB response mechanism. The defense response of chickpea to AB is a multifaceted venture which begins with recognition of pathogen leading to activation of a number of genes that further results in modifications in host cell wall, changes in ion flux through plasma membrane, formation of reactive oxygen species (ROS), distinctive biochemical changes and induced expression of various PR proteins, chitinases, dirigent proteins and GSTs. The defense mechanism involves a number of TFs like MYB, WRKY, ERF and signal transduction by various phytohormones like abscisic acid, salicylic acid and jasmonic acid (Figure 9). We have identified several TF encoding genes that showed differential expression under stress conditions. Several studies have reported the crucial role of various TFs in biotic stress response via gene regulatory networks (Park *et al*., 2001; Wang *et al*., 2009). In our study, genes encoding for NAC were induced in susceptible genotypes. NAC genes (*GhATAF1*) have been previously reported to increase cotton plant susceptibility to the fungal pathogens *Verticillium dahliae* and *Botrytis cinerea* (He *et al*., 2016). In *Arabidopsis*,* ATAF1* is known to negatively regulate defense responses against both necrotrophic fungal and bacterial pathogens by suppressing the action of PR proteins (Wang *et al*., 2009).

The formation of ROS, induction of PR proteins, biosynthesis of oxylipins and involvement of various phytohormone signaling pathways like abscisic acid, jasmonic acid mediated‐signaling pathways in AB response is supported by the GO enrichment analysis presented in the study. The ROS generated as a result of infection is known to be quenched by flavonoids such as flavanones and chalcones. The induced expression of chalcone‐flavanone isomerase, an important enzyme involved in the biosynthesis of chalcone at 3rd dpi in resistant genotypes as compared to susceptible genotypes was observed.

One of the most important class of defense genes is PR proteins which are known to exhibit antifungal activity against many plant pathogenic fungi such as, *Phytophthora infestans*,* Phytophthora parasitica* and *Uromyces fabae* (Matić *et al*., 2016). Another class of defense‐related genes, which are induced in plants under pathogen stress are peroxidases. Upon infection, peroxidases create a highly toxic environment by massive production of reactive oxygen and nitrogen species to prevent cellular diffusion of pathogens. In this study also, the resistant genotypes exhibited an increase in expression of various classes of PR proteins and peroxidases upon pathogen attack. Among the defense responsive genes, *Snakin‐2*, an antimicrobial peptide and DRRG49‐C were induced in resistant genotypes under stress conditions. These genes were also found to positively regulate defense responses against AB stress in previous studies (Coram and Pang, 2006; Mantri *et al*., 2010). Dirigent proteins are known to be involved in biosynthesis of lignan and are important for secondary metabolism and pathogen resistance (Li *et al*., 2017b). The induced expression of plant dirigent proteins in resistant genotypes as compared to susceptible genotypes might be an indication of their involvement in AB resistance. Aldo/keto reductase (AKR) is a large family known to be involved in plant defense and work by reducing aldehydes and ketones to their respective alcohols (Penning, 2004). AKRs were induced in resistant genotypes at 3rd dpi under stress indicating the important role of anti‐oxidation and detoxification in chickpea in response to AB (Figure 9). The up‐regulation of several defense responsive genes such as chitinase, osmotin and dirigent at 3rd dpi and their down‐regulation at 7th dpi in resistant genotypes might indicate that the resistant genotypes were able to combat infection by degrading the fungal cell walls and promoting lignan biosynthesis at an early stage of infection (3rd dpi). Similar results have also been reported in rice in response to fungal infection (Wang *et al*., 2014). The induced expression of senescence‐associated genes and genes related to ethylene and jasmonic acid signaling pathways in susceptible genotypes was observed at severe stress (7th dpi). The up‐regulation of gene coding for an allene oxide synthase which catalyzes the dehydration of the hydroperoxide to an unstable allene oxide in the jasmonic acid biosynthetic pathway was observed in susceptible genotypes. Various studies have suggested the role of ethylene and jasmonic acid in promoting leaf senescence (Kim *et al*., 2015). Also, it has been reported that jasmonic acid contributes to *Fusarium graminearum* susceptibility by attenuating the activation of SA signaling (Makandar *et al*., 2010). Thus, the higher expression of the jasmonic acid related genes in the susceptible genotypes, with respect to resistant genotypes, might contribute to their susceptibility. Interestingly, the induced expression of several defense‐responsive genes like NBS‐LRR and various PR proteins like chitinases and glucanases under control conditions suggest the possible existence of basic priming mechanism in resistant genotypes.

Since past few decades, the small non‐coding RNAs especially, the miRNAs have emerged as master modulators of gene expression at the post‐transcriptional level and are promising candidates for crop improvement (Tang and Chu, 2017). Using microarray and deep sequencing approaches, several stress‐responsive miRNAs have been identified in various crop plants (Candar‐Cakir *et al*., 2016; Guo *et al*., 2017). In chickpea also, a few studies reporting the genome‐wide discovery of miRNAs (Jain *et al*., 2014; Srivastava *et al*., 2015) and stress‐responsive miRNAs (Kohli *et al*., 2014) are available, but there is no study characterizing the role of miRNAs in AB resistance. In this study, we employed high‐throughput small RNA sequencing of a large number of samples under control and stress conditions to systematically study the effect of AB stress on different chickpea genotypes at different time points. By strictly adhering to criteria for annotation of plant miRNAs, the study identified a total of 651 miRNAs, of which 173 were novel. Several previous studies have reported that conserved/known miRNAs are important for various developmental processes, whereas the recently evolved or evolving novel miRNAs might be important for species‐specific gene regulatory functions (Srivastava *et al*., 2015; Sunkar *et al*., 2008). The features of identified miRNAs like length, GC content and the abundance of 5′‐uridine residue were in concordance with the previous studies (Jain *et al*., 2014; Kohli *et al*., 2014).

A total of 297 miRNAs demonstrated differential expression patterns in at least one sample/condition and it was seen that a significant number of novel miRNAs were also a part of these differentially expressed miRNAs. Despite the similarity, the members of same miRNA family respond differently to AB stress. It was seen that the different isoforms of the same miRNA family exhibited different expression patterns. For example, miR171 showed an up‐regulation at 3rd dpi and down‐regulation at 7th dpi under stress whereas its isoform miR171b showed the opposite trend of down‐regulation at 3rd dpi and up‐regulation at 7th dpi in resistant genotypes. Similar events were also reported in other plant species such as soybean where different members of miR396 family showed different expression patterns under stress (Fang *et al*., 2013). The function of miRNA can be inferred by accurately identifying their targets. The miRNA targets can be predicted both computationally and by the use of sequencing techniques like degradome sequencing which gives the pattern of RNA degradation. A very high number of targets (2131) were predicted for the identified miRNAs by using both these approaches. Interestingly, the GO enrichment analysis of these targets highlighted that these targets were mainly involved in regulating diverse developmental processes and various components of a fungal stress response. For instance, miR160 was seen to target auxin response factors which are important components of auxin signaling pathway involved in plant growth and development. Similarly, a member of miR482 and miR171 family was found to target NBS‐LRR and GST gene, respectively, both of which are involved in defense response.

The accumulation of miRNA, in general, leads to down‐regulation of their target genes and vice‐versa. The integrated analysis of the differentially expressed miRNAs and mRNAs revealed several AB responsive miRNAs. The up‐regulation of various stress‐responsive miRNAs like miR482b‐3p, miR159k‐3p, nov_miR66 and miR171 and the down‐regulation of their targets, NBS‐LRR, PR protein, serine‐threonine kinase and PPR protein, respectively, in susceptible genotypes compared to their resistant counterparts suggests that miRNA regulated resistance might be nurtured during AB infection. It is speculated that in susceptible genotypes, the disease resistance genes might have undergone post‐transcriptional silencing by their respective miRNAs and hence, were not able to combat infection. The degradome analysis also confirmed the cleavage of these genes by their respective miRNAs. The members of miR482 family were also found to target NBS‐LRR genes in previously reported studies (Shivaprasad *et al*., 2012; Zhu *et al*., 2013). In the present study, miR162 was found to target a Dicer‐like gene (*Ca_01367*) and it was observed that in susceptible genotypes, the expression of miR162 increased and the expression of its target decreased in response to AB infection. The decrease in the expression of *Ca_01367* in susceptible genotypes under AB infection was also reported in a previous study (Garg *et al*., 2017). Under stress, the repressed expression of miR319l and the induced expression of its target TEOSINTE BRANCHED/CYCLOIDEA/PCF (TCP) was observed in susceptible genotypes (Figure 9). It has been previously reported that miR319l alters the expression of TCP which further modulates the expression of jasmonic acid biosynthesis genes, thus, positively regulating leaf senescence (Kim *et al*., 2015).

In summary, the present study is the first attempt to integrate mRNA and miRNA expression data along with degradome analysis to identify key regulatory miRNA‐target circuits in chickpea in response to AB. The study highlighted more extensive genotype‐specific response to AB stress than the response common to both the resistant and susceptible genotypes. The study identified a number of genes showing similar expression in two resistant genotypes, ICCV 05530 and ILC 3279 but different in BC_3_F_6_ line. The expression of these genes in BC_3_F_6_ line was similar to its recurrent parent C 214. The use of introgression line (BC_3_F_6_) in the study helped to pinpoint the genes which are most likely to be implicated in resistance. The genes showing similar expression pattern in all resistant genotypes including BC_3_F_6_ line could be considered as candidates which might play an important role in AB resistance. The study also highlighted that the similar set of genes exhibited different expression patterns at different stages of infection. The integrated analysis of miRNA and RNA sequencing identified 12 miRNA‐mRNA pairs which exhibited contrasting expression in resistant and susceptible genotypes and included genes like NBS‐LRR, PR proteins and miRNAs like miR482b‐3p and miR159k‐3p. Five out of these 12 pairs have been validated by degradome sequencing and few genes of these pairs are present in previously reported AB resistance QTLs. Overall, these genes and miRNAs give a clear indication of miRNA mediated stress response in chickpea in response to AB stress and can be considered as candidates for developing resistance in chickpea against AB using both breeding or genetic engineering approaches. The comprehensive datasets of differential expression patterns of genes and miRNAs in different chickpea genotypes present new insights into the resistance mechanism of chickpea to AB stress. These datasets will also serve as a valuable resource to investigate transcriptional reprogramming in chickpea in response to other fungal infections.

## Materials and methods

### Plant material, stress treatment and RNA extraction

A total of four chickpea genotypes and an introgression line with contrasting phenotype for AB stress (C 214 and Pb 7 – susceptible and ILC 3279, ICCV 05530 and BC_3_F_6_ – moderately resistant) were used in the study (Garg *et al*., 2017; Pande *et al*., 2011; Varshney *et al*., 2014). BC_3_F_6_ (ICCX‐100176‐470‐2‐7) line is an introgression line obtained by introgression of AB resistance QTLs (ABQTL‐I and ABQTL‐II) from ILC 3279 into genetic background of C 214, an elite chickpea cultivar (Varshney *et al*., 2014).

For the imposition of stress, seedling raising and inoculum preparation were performed as described earlier (Pande *et al*., 2011). Ten‐day‐old seedlings of above‐mentioned genotypes, acclimatized for 24 h to 20 ± 1 °C temperature and 12 h of photoperiod in controlled environment facility, were sprayed with the *A. rabiei* spore suspension of 5 × 10^4^ conidia/mL until runoff. The aerial tissues of seedlings were harvested on 3rd and 7th dpi in a set of three biological replicates each. Along with the AB inoculated samples, control (non‐inoculated) samples were also harvested from both the time points. From previous studies, it is known that early response of infection initiates at 24–48 h post inoculation and prevails until 7th dpi after which plants react defensively to the AB infection causing tissue decline (Pande *et al*., 2005; Sharma and Ghosh, 2016). Considering this, 3rd and 7th dpi were selected for the current study. The tissues were stored at −80 °C until RNA isolation. Total RNA from aerial tissues of seedlings was isolated using “NucleoSpin^®^ RNA Plant” kit (Macherey‐Nagel, Düren, Germany) according to the manufacturer's instructions. The qualitative and quantitative assessment of these total RNA samples were conducted using Agilent 2100 Bioanalyzer (Agilent Technologies, Santa Clara, CA) and Nanodrop 8000 Spectrophotometer (Thermo Fisher Scientific, Waltham, MA). The total RNA from each tissue was used for library construction for transcriptome, small RNA and degradome sequencing. The library construction and sequencing were conducted by BGI‐Shenzhen, China.

### Transcriptome sequencing and data pre‐processing

The high‐quality total RNA (RIN ≥ 8) was used for transcriptome library construction using TruSeq RNA Sample Prep Kit v2 (Illumina Inc., San Diego, CA) according to manufacturer's instructions. Briefly, mRNA was isolated from total RNA using oligo‐dT magnetic beads and fragmented, followed by cDNA synthesis using SuperScript II Reverse Transcriptase (Invitrogen, Carlsbad, CA). The cDNA fragments were amplified to generate transcriptome libraries which were sequenced on Illumina HiSeq 4000 platform using 100 bp paired‐end strategy. In total, 20 samples representing 5 genotypes (ILC 3279, ICCV 05530, C 214, Pb 7 and BC_3_F_6_) under control and AB stress conditions at two time points (3rd and 7th dpi) were subjected to transcriptome sequencing. The raw reads obtained from sequencing all these samples were processed to remove primer/adaptor contamination and low‐quality reads (>20% of the bases with a phred quality score < 10) using Trimmomatic v0.35 (Bolger *et al*., 2014).

### Identification of DEGs and their co‐localization with QTLs

The filtered reads were mapped on the chickpea reference genome v1.0 (Varshney *et al*., 2013) using Tophat2 (Kim *et al*., 2013). The mapped reads from each sample were assembled using Cufflinks v2.2.1 (Trapnell *et al*., 2012) to generate reference‐guided assemblies which were further merged to generate a consensus assembly using Cuffmerge. The consensus assembly thus obtained was used for downstream analysis. The genes identified in the assembly were studied for their expression patterns across all samples using Cuffdiff. DEGs were identified between different samples under control (non‐inoculated) and AB inoculated conditions at different time points. A gene was considered to be differentially expressed between two samples if it exhibited log_2_ fold change value of ≥2 or ≤–2. For co‐localization studies, the information of the markers associated with AB resistance QTLs was retrieved from cool season food legume database. The sequence similarity search (blastn) using primer sequences of these markers as query against the reference genome was performed to obtain physical locations of markers. The blastn hits with 100% coverage of both query and subject were only selected. The co‐localization between the identified DEGs and physical positions of AB resistance QTLs was further analysed using Microsoft Excel.

### Annotation and GO enrichment of DEGs

The putative functions of the identified DEGs were determined by subjecting the DEGs to blastx similarity searches (*E*‐value 1e‐05) against UniProtKB, Swiss‐Prot and NCBI non‐redundant protein database. The GO enrichment analysis to identify the over‐represented functional categories was carried out using R‐based GOseq package (Young *et al*., 2010). The GO terms with corrected *P*‐value ≤ 0.05 were considered to be significantly enriched for a given set of genes and were further clustered to view the most interconnected categories using Cytoscape. The DEGs were mapped to their respective pathways using KEGG Automatic Annotation Server (Moriya *et al*., 2007). Pathway enrichment analysis was done based on hypergeometric model (Boyle *et al*., 2004) with a significance threshold of *P*‐value 0.05.

### Small RNA sequencing and data pre‐processing

Small RNA libraries were constructed using the Illumina TruSeq Small RNA Library Prep Kit (Illumina Inc., San Diego, CA) according to the manufacturer's instructions. In brief, 1 μg of total RNA from each sample was separated by polyacrylamide gel electrophoresis. RNA fragments of length 18–30 nt were enriched and were ligated to 3′ and 5′ adapter using T4 RNA ligase. The adaptor‐ligated RNA molecules were then subjected to cDNA synthesis, followed by amplification and sequencing on Illumina HiSeq 4000. The raw reads obtained from sequencing were processed for various quality controls which included removal of low‐quality reads, reads with adaptor, primer contamination and poly A tail using Trimmomatic v0.35. The reads shorter than 18 nt and longer than 35 nt were discarded. The clean reads from each sample were further screened against rRNA, tRNA, snoRNA and repeat sequences. After filtering, the redundant reads were processed into unique sequences with associated read counts for miRNA prediction.

### Identification of known and novel miRNA

The filtered unique reads from each sample were mapped onto the plant miRNAs from miRBase (release 21; Kozomara and Griffiths‐Jones, 2014) for identification of conserved miRNAs. The alignment was done using Bowtie alignment tool v1.1.2 with two mismatches and the unaligned unique reads were further used for novel miRNA prediction. These remaining unique reads were mapped on chickpea genome using Bowtie with no mismatch and for aligned reads, putative precursor sequences of 250 bp were extracted. From the identified precursors, novel miRNAs were identified using miRDeep‐P, a probabilistic model‐based miRNA prediction software especially designed for plant miRNAs (Yang and Li, 2011). The novel prediction in miRDeep‐P revolves around the secondary structure, presence of 3′‐overhang, star miRNA evidence, less than six nucleotides difference between mature and star miRNA lengths, the Dicer cleavage site and the minimum free energy of the small RNA reads (Meyers *et al*., 2008). Further, on the basis of sequence similarity, the identified miRNAs were clustered into families using CD‐HIT (Fu *et al*., 2012) with 90% identity. Subsequently, the mRNA targets of identified miRNAs were predicted using psRNATarget server (Dai and Zhao, 2011) with default parameters.

### Expression analysis of miRNAs

For determining the expression of identified miRNAs in each sample, R based DESeq2 (Love *et al*., 2014) package was used. A normalization factor calculated by DESeq2 was used to normalize the raw read counts of miRNA followed by identification of differentially expressed miRNA. A miRNA was considered to be significantly differentially expressed between the two samples if it exhibited a log_2_ fold change ≥1 or ≤−1 and a *P*‐value ≤ 0.05.

### Degradome library construction and analysis

For degradome library construction, total RNA from 3rd and 7th dpi control and stress samples were pooled together to generate two libraries for each genotype, one representing control and other stress. For four genotypes and one introgression line, a total of 10 libraries were generated. The library construction was performed as previously described by German *et al*. (2008) with some modifications followed by sequencing. The raw reads (single‐end; 50 bp) were processed to remove low‐quality reads, reads with ‘N's and any reads with adaptor and primer contamination using Trimmomatic v0.35. The filtered reads were searched against all other non‐coding RNA sequences from Rfam except miRNA using Bowtie. The reads aligning to rRNAs, tRNAs, snoRNAs and repeats were removed. The clean reads thus obtained were mapped on the chickpea transcriptome (Varshney *et al*., 2013) with maximum one mismatch. The reads mapping to the sense strand of transcriptome were processed using CleaveLand v4.4 pipeline (Addo‐Quaye *et al*., 2009) to predict miRNA cleavage sites. The cleavage sites at 10th position relative to the aligned miRNA and with *P*‐value ≤ 0.05 were considered as significant. The identified sites were categorized into five categories (0–4) based on the read abundance at that position. Categories 0–3 have more than one read mapped at the cleavage site and category 4 has only one read. These different categories indicate the confidence level of prediction with category 0 with maximum confidence and category 4 with minimum confidence.

### Validation of DEGs and miRNAs

For validation of the gene and miRNA expression obtained from high‐throughput sequencing, qRT‐PCR of randomly selected genes and miRNAs was performed. The qRT‐PCR analysis was carried out for 20 samples which included control (non‐inoculated) and AB inoculated samples of resistant (ILC 3279, ICCV 05530 and BC_3_F_6_) and susceptible (C 214 and Pb 7) genotypes at two time points (3rd and 7th dpi). The primers including miRNA‐specific stem‐loop RT, forward primers and universal reverse primer for the selected miRNAs were designed according to Kramer (2011). Further, the gene‐specific primers were designed using BatchPrimer3 v1.0 (You *et al*., 2008). The qRT‐PCR reactions were performed using SYBR green master‐mix in 96 well‐plates with three biological replicates and two technical replicates using Glyceraldehyde 3‐phosphate dehydrogenase (GAPDH) as the endogenous control. The PCR conditions used were as follows: 2 min at 50 °C, 10 min at 95 °C, and 40 cycles of 15 s at 95 °C and 1 min at 60 °C. The relative transcriptional levels in terms of fold‐change were determined using the 2^−ΔΔCt^ method and Student's *t*‐test was used to calculate significance (Livak and Schmittgen, 2001).

## Conflict of interest

The authors declare that they have no competing interests.

## Supporting information


**Figure S1** Length distribution of unique small RNAs in twenty small RNA libraries.
**Figure S2** The 5′ nucleotide composition of identified miRNAs in chickpea and other species.
**Figure S3** Gene ontology enrichment of miRNA targets.
**Figure S4** Pathway annotation of targets using KEGG database.
**Figure S5** T‐plots and miRNA‐mRNA alignments validated by degradome sequencing where (a) miR157a cleaves Senescence‐associated (*Ca_15107*) gene; (b) miR5232 cleaves calcium‐transporting ATPase (*Ca_12185*) gene.
**Figure S6** Validation of high‐throughput sequencing through qRT‐PCR (a) RNA‐seq (b) Small RNA‐seq where ‘*r*’ is the correlation coefficient between high‐throughput sequencing data and qRT‐PCR data

**Table S1** Details of identified miRNAs (known and novel), their targets and annotation.
**Table S2** Statistics of degradome sequencing and splicing sites identified in each sample.
**Table S3** List of primers used for qRT‐PCR experiments.

## Data Availability

The sequencing data have been deposited in NCBI Sequence Read Archive (SRA, http://www.ncbi.nlm.nih.gov/sra) with the BioProject ID PRJNA479940.
