# Supplementary material for: Integrated transcriptome, small RNA and degradome sequencing approaches provide insights into Ascochyta blight resistance in chickpea
Source: Plant Biotechnol J. 2018 Dec 1;17(5):914–31. doi: 10.1111/pbi.13026 (PMC6472043; doi:10.1111/pbi.13026)
Supplement: Supplementary file 1 — Figure S1 Length distribution of unique small RNAs in twenty small RNA libraries. Figure S2 The 5′ nucleotide composition of identified miRNAs in chickpea and other species. Figure S3 Gene ontology enrichment of miRNA targets. Figure S4 Pathway annotation of targets using KEGG database. Figure S5 T‐plots and miRNA‐mRNA alignments validated by degradome sequencing where (a) miR157a cleaves Senescence‐associated (Ca_15107) gene; (b) miR5232 cleaves calcium‐transporting ATPase (Ca_12185) gene. Figure S6 Validation of high‐throughput sequencing through qRT‐PCR (a) RNA‐seq (b) Small RNA‐seq where ‘r’ is the correlation coefficient between high‐throughput sequencing data and qRT‐PCR data [file PBI-17-914-s002.pdf]

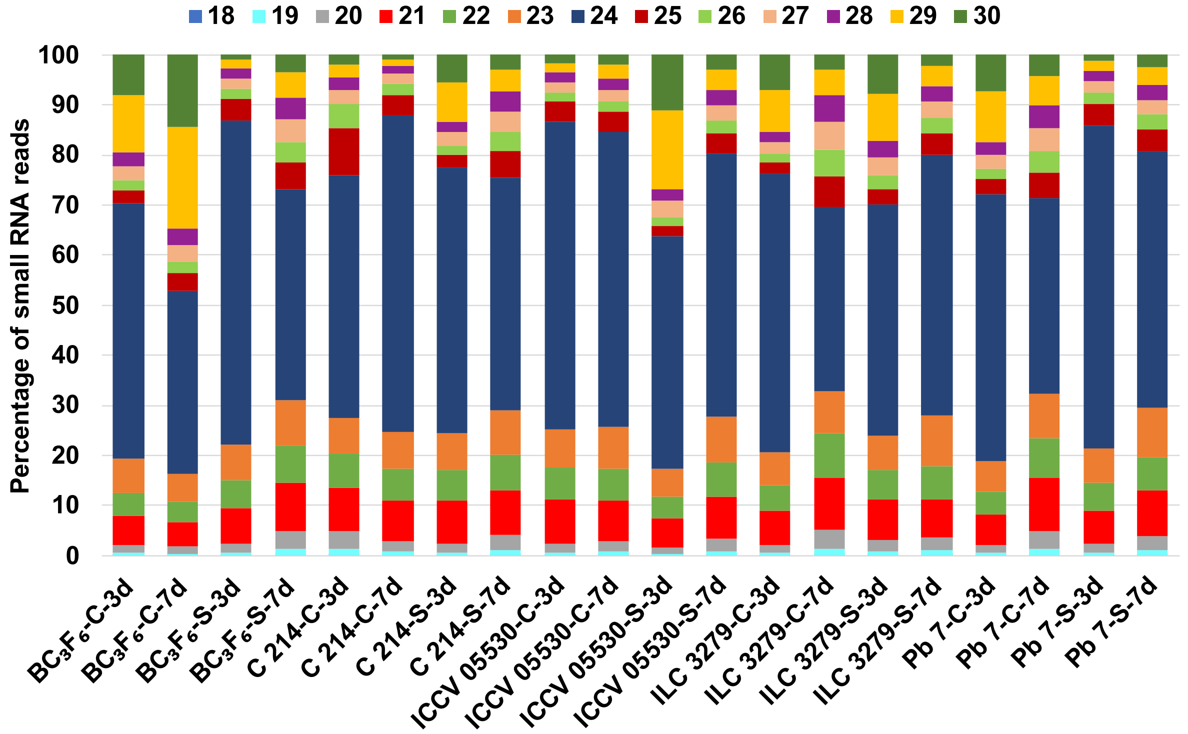

Figure S1. Length distribution of unique small RNAs in twenty small RNA libraries. Small RNAs with length between 21 and 24 nt account for the majority of the reads and 24 nt is the largest class.

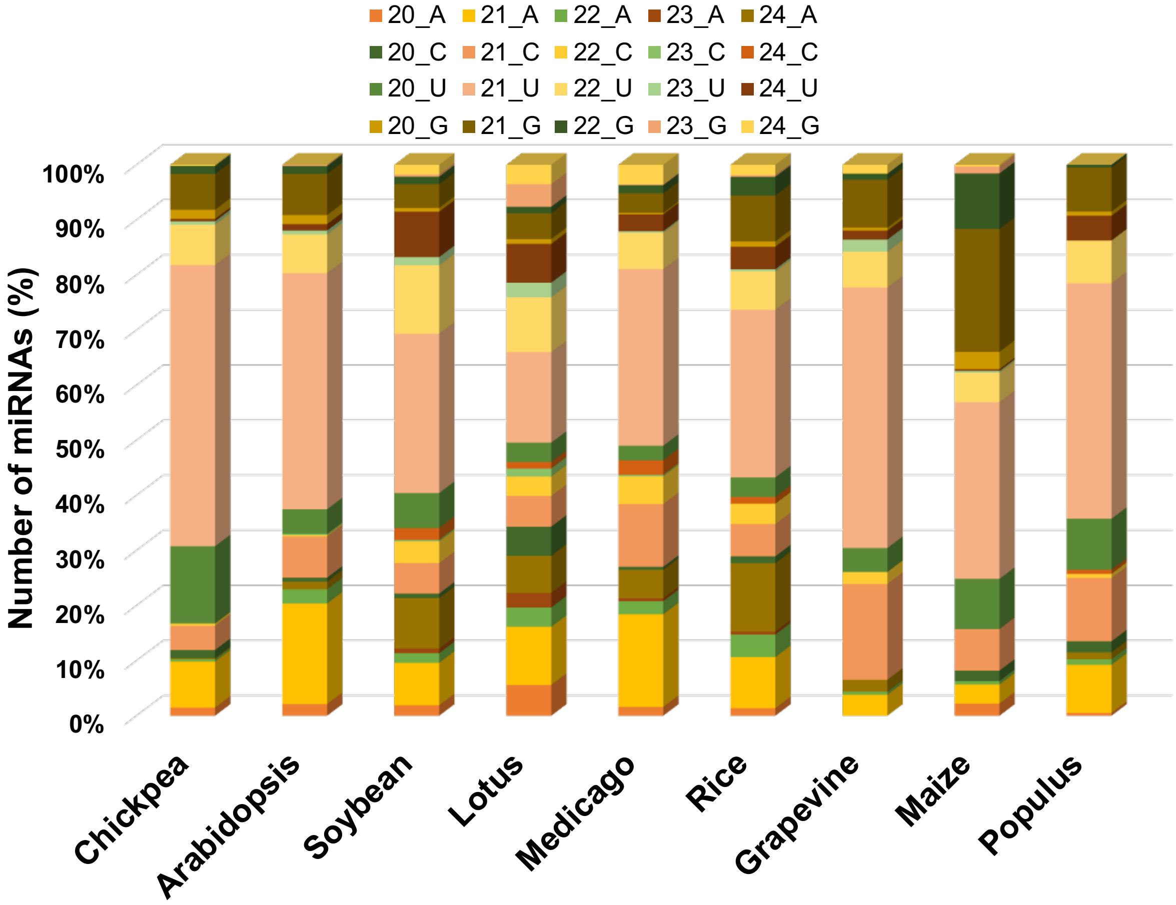

Figure S2. The 5’ nucleotide composition of identified miRNAs in chickpea and other species.

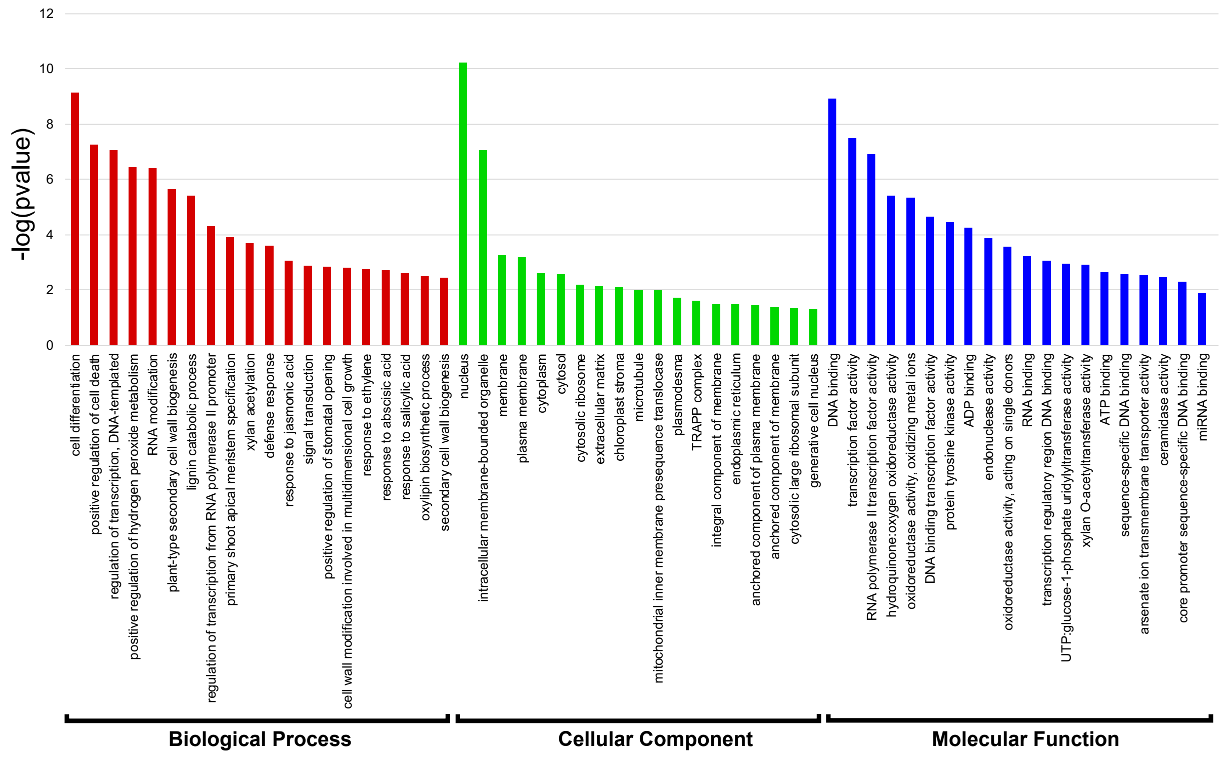

Figure S3. Gene ontology enrichment of miRNA targets. Bar graphs represent top enriched GO terms of miRNA targets in three categories: biological processes, molecular functions, and cellular components.

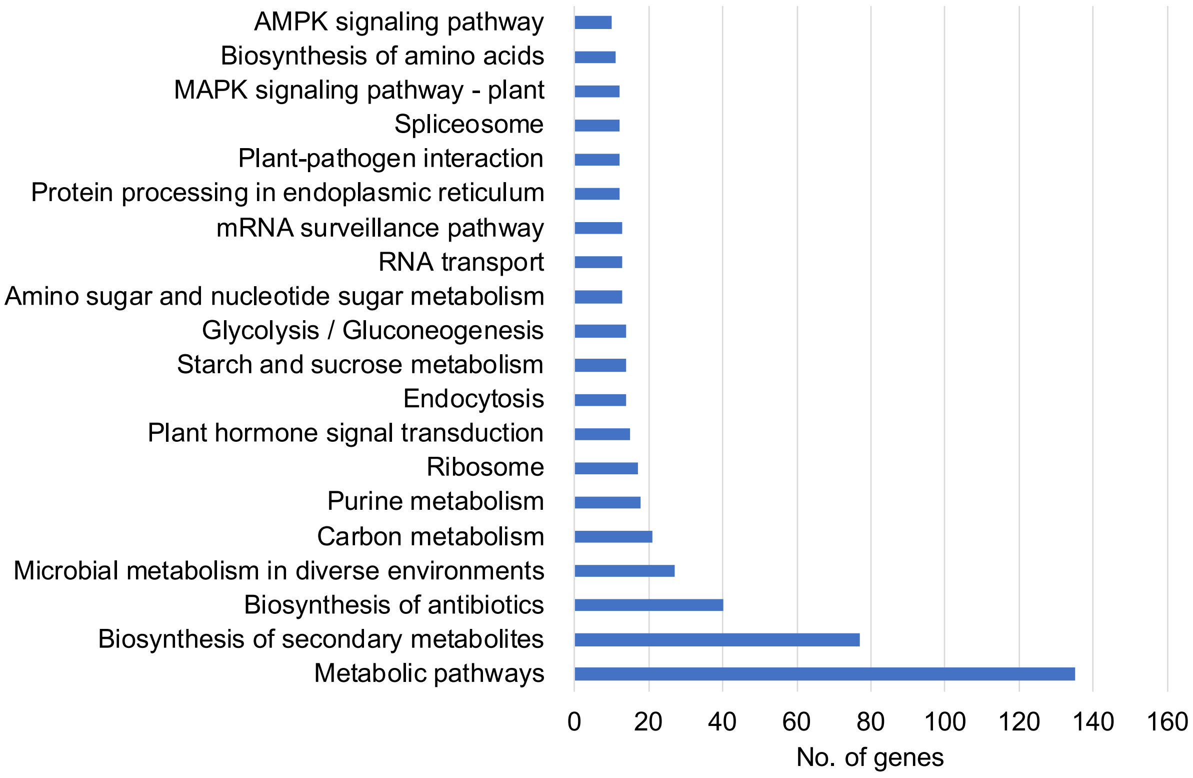

Figure S4. Pathway annotation of targets using KEGG database. The most abundant pathways are shown.

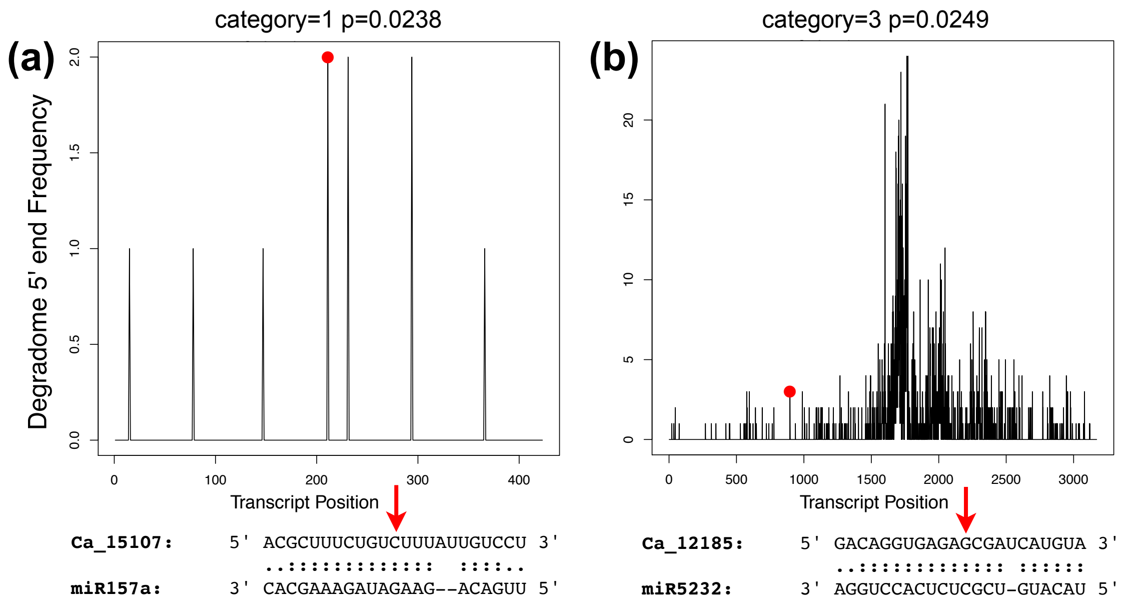

Figure S5. T-plots and miRNA-mRNA alignments validated by degradome sequencing where (a) miR157a cleaves Senescence-associated (*Ca_15107*) gene; (b) miR5232 cleaves calcium-transporting ATPase (*Ca_12185*) gene. The red dots and arrows represent the cleavage nucleotide positions on the target genes.

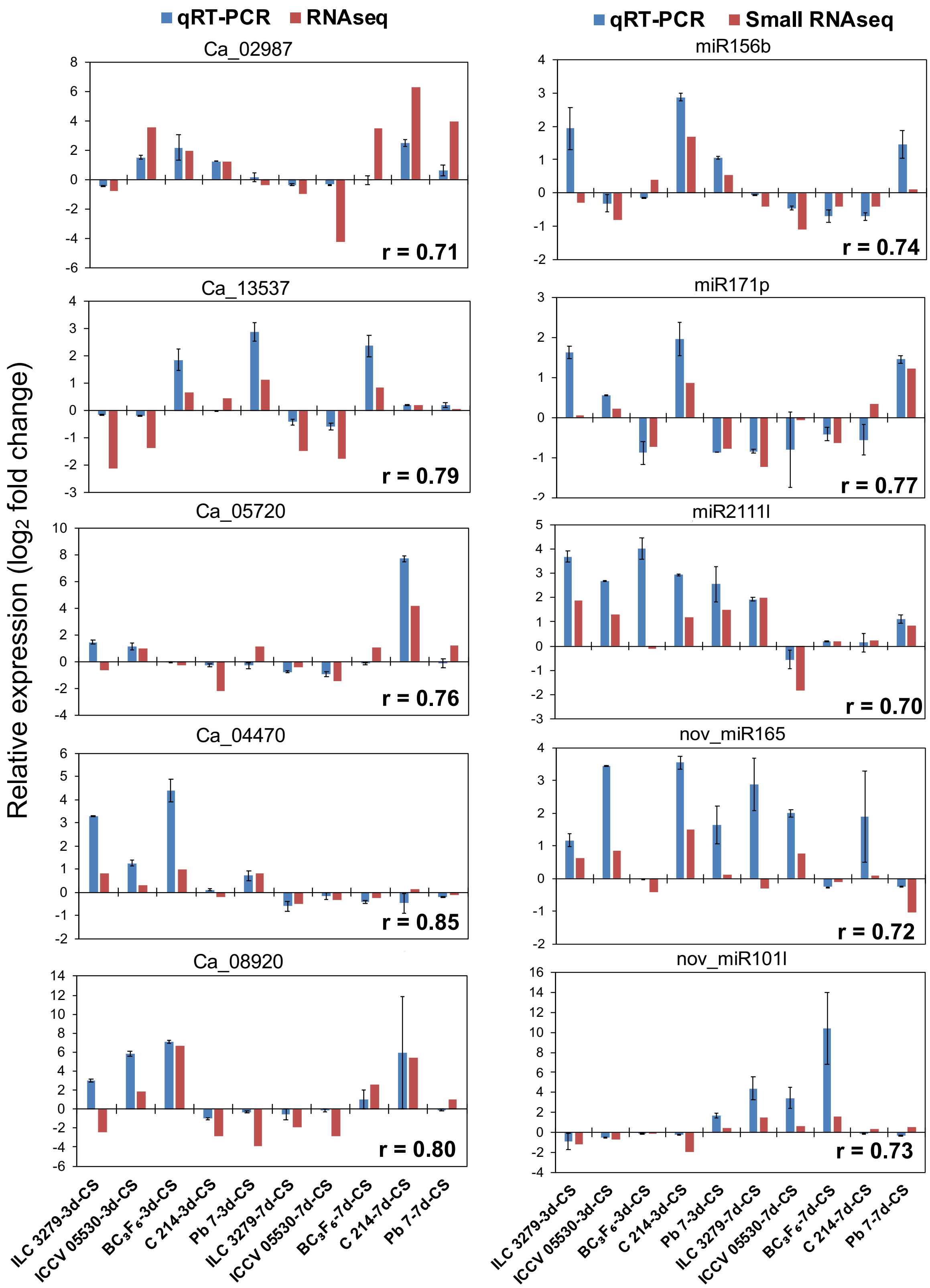

Figure S6. Validation of high throughput sequencing through qRT-PCR (a) RNA-seq (b) Small RNA-seq where 'r' is the correlation coefficient between high throughput sequencing data and qRT-PCR data. The bars show the relative expression (log_2 fold change) of different genes and miRNAs under control and stress conditions across different genotypes at both 3rd and 7th dpi.
